# Machine learning-based prediction model for drug target identification and MASH improvement: a comprehensive analysis of biochemical and ferroptosis/autophagy biomarkers

**DOI:** 10.1007/s13105-026-01181-3

**Published:** 2026-04-27

**Authors:** Marwa Matboli, Aly Elanwar, Radwa Khaled, Abdelrahman Khaled, Eman Hamdy Badr Eltantawy, Ghada Galal Hamam, Manar Yehia Ahmed, Manar Fouad, Ahmed Asmaa Tarek, Maryam Elmasry, Marwa M El-Shafei, Basma Emad Aboulhoda, Gouda Ibrahim Diab, Ibrahim H. Aboughaleb

**Affiliations:** 1 Medical biochemistry and molecular biology department, Faculty of Medicine, Ain Shams University, Cairo, Egypt; 2 General Surgery Department, Faculty of medicine, Ain Shams University, Cairo, 11566 Egypt; 3 Translational and applied Science hub, Faculty of medicine, Ain Shams University, Cairo, 11566 Egypt; 4 Bioinformatics Group, Center of Informatics Sciences (CIS), school of information technology and computer sciences, Nile University, Giza, Egypt; 5 Department of Histology, Faculty of Medicine, Ain-Shams University, Cairo, Egypt; 6 Medical Physiology, faculty of medicine, Ain shams university, Cairo, Egypt; 7 Pathology Department, Faculty of Oral and Dental Medicine, Misr International University, Cairo, Egypt; 8 Anatomy and Embryology department, Faculty of Medicine, Cairo University, Cairo, Egypt; 9 Biomedical Engineering Department, Egyptian Armed Forces, Cairo, Egypt

**Keywords:** Nonalcoholic steatohepatitis, Ferroptosis, Autophagy, Machine learning, Drug targets

## Abstract

Metabolic dysfunction-associated steatotic liver disease (MASLD), marked by excess fat in the liver, has become the most prevalent chronic liver disease worldwide, affecting over 30% of adults. Its advanced form, metabolic dysfunction associated steatohepatitis (MASH), includes liver ballooning, and inflammation, and can progress to cirrhosis and hepatocellular carcinoma (HCC). Despite the increasing burden, effective pharmacotherapies for MASLD/MASH are still lacking. Programmed cell death mechanisms, such as autophagy and ferroptosis, are critical in the pathology of MASLD, influencing liver inflammation, fibrosis, and malignant transformation. This study employed six machine learning models—Random Forest, Logistic Regression, Extra Trees Classifier, Linear Discriminant Analysis, and Light Gradient Boosting Machine—to identify significant drug targets using Febuxostat, Perindopril, Amlodipine, and Atorvastatin, evaluated through molecular, biochemical, immunohistochemical, and pathological markers. We identified genes associated with MASH using microarray datasets from the Gene Expression Omnibus database, followed by protein-protein interaction and functional enrichment analyses to select genes related to ferroptosis, autophagy, and their epigenetic regulators (miRNAs-LncRNAs) in MASH-induced rats. Quantitative real-time PCR validated the expression of selected networks (mRNAs-miRNAs-LncRNAs). Additionally, we measured biochemical, inflammatory, and liver pathology markers to ensure the model’s robustness. Our results identified 16 out of 29 valuable therapeutic targets with an accuracy of 88.74% and an AUC of 0.9745, including LPCAT3, HGS, TSG101, SNF8, rno-miR-27a-5p, rno-miR-329-5p, CTBP1-AS2, ALT, AST, ALP, GGT, D. Bilirubin, Albumin, TMAO, GPX4, and TGFβ1.

**Supplementary information:**

The online version contains supplementary material available at 10.1007/s13105-026-01181-3.

## Introduction

The recent nomenclature updates from NAFLD “Non-alcoholic fatty liver disease” to MAFLD “metabolic associated fatty liver disease”, and subsequently to MASLD “metabolic dysfunction-associated steatotic liver disease”, underscore the importance of more precisely defining the condition’s underlying causes, given its high prevalence of up to a quarter adult worldwide [1, 2]. This change marks a significant shift in understanding, emphasizing the complex relationship between metabolic syndrome and liver health. MASLD is characterized by liver steatosis combined with at least one of five cardiometabolic risk factors associated with metabolic syndrome, such as impaired glucose regulation, type 2 diabetes (T2D), obesity, hypertension, or dyslipidemia [3–5]. Metabolic dysfunction associated steatohepatitis (MASH) pathologically is characterized by steatosis along with hepatocyte ballooning and lobular inflammation, which together form the ‘necroinflammatory’ disease activity. MASH is a major factor in the progression of cirrhosis and hepatocellular carcinoma (HCC) [6, 7].

Ferroptosis is a unique, regulated form of cell death, characterized by the iron-dependent buildup of lipid peroxidases, and its induction in hepatic stellate cells (HSCs) reduces liver fibrosis [8]. This process involves various cellular metabolic pathways, including iron management, lipid metabolism, mitochondrial function, redox balance, and membrane repair [9]. Lipid peroxidation, facilitated by both enzymatic pathways and the enzyme-independent Fenton reaction, is directly implicated in ferroptosis. A critical event in ferroptosis is the incorporation of polyunsaturated fatty acids (PUFAs) into phospholipids by acyl-CoA synthetase long-chain family member 4 (ACSL4) [10]. Blocking lipid peroxidation, either pharmacologically or genetically, significantly prevents ferroptosis [11]. Autophagy, a cellular degradation process, plays a dual role in maintaining cellular homeostasis and is thus intimately linked to the development, progression, and prognosis of MASLD [12]. It often removes unnecessary proteins or organelles, recycling them to support cell survival, however, excessive or dysregulated autophagy can also lead to cell death [13]. Increasing evidence indicates that autophagic flux is heightened during ferroptosis, and inhibiting autophagy can prevent ferroptotic cell death [14].

Despite the unmet need for MASH treatments, there are currently no approved drugs available [15]. However, the ideal approach for drug development in MASH involves targeting both steatohepatitis and fibrosis while improving cardiometabolic risk factors [16]. When developing new drugs for MASH, it is crucial to assess their impact not only the liver disease but also on related conditions like T2D and cardiovascular disease [17]. It is unlikely that a single agent can effectively address all aspects of this multifaceted liver disease. Considering the complexity of MASH, it is highly probable that multiple classes of drugs targeting different mechanisms will be required [18].

Recent studies have shown a potential link between metabolic syndrome (including MASLD or MASH) and hyperuricemia [19]. Xanthine oxidase, an enzyme present in various organs such as the liver, kidney, and vascular tissue, plays a role in the production of uric acid (UA) and generates reactive oxygen species (ROS) during the process [20]. Febuxostat, an inhibitor of xanthine oxidase (XO), has demonstrated a strong protective effect against MASH progression [21]. By reducing uric acid levels and inhibiting oxidative stress, XO inhibitors like Febuxostat may have therapeutic benefits in preventing and treating MASH. The renin-angiotensin system (RAS) is reported to be activated in individuals with chronic liver diseases like cirrhosis [22]. Angiotensin-II (AT-II) has been shown to induce HSC contraction and proliferation [23]. In terms of oxidative stress, AT-II stimulates NADPH oxidase, leading to substantial hepatic ROS production, lipid peroxidation, inflammatory cell infiltration, apoptosis, and fibrosis [24]. Clinical studies have demonstrated that angiotensin-converting enzyme (ACE) inhibitors, such as Perindopril, and AT1 receptor blockers (ARBs) can significantly reduce the development of experimental liver fibrosis and suppress activated HSCs [25]. Amlodipine, a third-generation dihydropyridine calcium channel blocker, functions by inhibiting calcium entry into vascular smooth muscle and myocardial cells, thereby lowering peripheral vascular resistance [26]. Earlier research found that amlodipine diminished Kupffer cell activation, enhanced hepatocyte viability, and protected liver cells from superoxide-induced injury in hypertensive rats [27]. For instance, the statin medication Atorvastatin, which works by inhibiting the 3-hydroxy-3-methylglutaryl-coenzyme A reductase enzyme, has been shown to improve NAFLD activity scores and reduce elevated transaminase levels in affected individuals [28, 29].

The field of epigenetics, which examines inheritable changes in gene expression without altering the DNA sequence, offers a new perspective on the development of MASLD/MASH. Epigenetic modifications play a crucial role in regulating various processes, including lipid metabolism, insulin sensitivity, mitochondrial function, and redox balance [30]. These epigenetic mechanisms involve the regulation of RNA processing, stability, and translation through the specific binding of small RNA molecules, such as microRNAs (miRNAs) and long non-coding RNAs (LncRNAs) [31].

Currently, there remains a lack of widely utilized biomarkers that can be extensively employed in clinical practice for the diagnosis and prognosis of MASH. Therefore, it is necessary to discover sensitive and specific markers closely linked to MASH to enhance early detection and treatment. In this study, we employed bioinformatics and machine learning (ML) techniques to identify the key biomarkers for MASH. We examined four potential drugs (Febuxostat, Perindopril, Amlodipine, and Atorvastatin) and identified target biomarkers associated with MASH from routine laboratory markers, as well as ferroptosis- and autophagy-related mRNAs, miRNAs, and lncRNAs. These findings were validated histologically using the non-alcoholic steatohepatitis activity score (NAS) to ensure their clinical reliability.

## Materials and methods

### Chemicals and drugs

Cholesterol and cholic acid were acquired from Ralin BV (Lijinbaan, Netherlands). Amlodipine was obtained from EPICO Company in Cairo, Egypt. Febuxostat was supplied by Eva Pharma Company (Cairo, Egypt). Perindopril was purchased from Global Napi Pharmaceutical Industries (Giza, Egypt). Atorvastatin was procured from Pfizer company (Cairo, Egypt).

### Experimental design

A hundred and forty male Wistar rats were procured from the Scientific Research Centre at Ain Shams University “MASRI”, weighing 150–180 gm, and were accommodated under ambient temperatures (20 ± 2) °C, 12/12-h light/dark cycle, and fed ad libitum. All animal experiments were performed under the approval of the Animal Ethics Committee of the Faculty of Medicine, Ain Shams University, Egypt((FMASU R 111/2022)). After a one-week acclimatization period, the rats were randomly assigned to different groups. (Ⅰ) the control group (*n* = 35); rats were provided with a standard chow diet. (Ⅱ) The MASH model group (*n* = 35): rats were fed with high sucrose and high-fat diet (HSHFD) for 12 weeks. (Ⅲ) Febuxostat subdivided into 3 groups (*n* = 10 each); rats were fed HSHFD for 12 weeks and administered (1.5, 3, and 6 mg/kg/day Febuxostat) (Ⅳ) Amlodipine (*n* = 10); rats were fed HSHFD for 12 weeks and administered amlodipine (1 mg/kg/day), (Ⅴ) Perindopril group (*n* = 10); rats were fed HFHSD for 12 weeks and administered Perindopril (1 mg/kg/day), (Ⅵ) Amlodipine/Perindopril group; rats were fed HFHSD for 12 weeks and administered with a combined (1 mg/kg/day. of amlodipine + 1 mg/kg/day of perindopril) and (Ⅶ) Atorvastatin-20; rats were fed HFHSD for 12 weeks and administered with (20 mg/kg/day atorvastatin). The drug administration was done through gastric gavage daily during the last 4 weeks of the study. At the end of the experiment, all the rats were anesthetized with urethane (1.2 g/kg; intraperitoneal injection), and their blood samples were collected from the retroorbital vein. The blood samples were centrifuged at 5000 rpm for 20 min to separate the serum, while their liver tissues were dissected through an abdominal incision, weighed, and frozen at −80 °C for immunohistochemical protein assessments, and RNA extraction. The right lobes were promptly preserved in a 10% neutral buffered formalin to facilitate subsequent histopathological examinations. Liver samples fixed in buffered formalin were processed for light microscopy by dehydrating in ethanol, embedding in paraffin, and slicing into 6-µm thick sections. Hematoxylin-eosin (HE) staining was used to evaluate histological features of steatohepatitis, while Masson’s Trichrome staining identified collagen fibers, and Sirius red (for demonstration of collagen fibers that appeared red). The MASH activity score (NAS) was determined according to the MASH Clinical Research Network (MASH CRN) scoring system by adding the scores for steatosis, lobular inflammation, and hepatocyte ballooning in HE-stained sections. The NAS ranges from 0 to 8, calculated, and graded as follows; MASH (Score ≥ 5), borderline (3–4), or no MASH (< 3). The stages of hepatocyte steatosis were determined based on the percentage of hepatocytes containing intracytoplasmic fat droplets. The degree of steatosis was graded on a four-point scale: score 0 (< 5%, none), score 1 (5–33%, mild), score 2 (> 33–66%, moderate), and score 3 (> 66%, severe). Intralobular inflammation was graded based on the number of inflammatory foci observed per 20× magnification. It was graded as 0 (none), 1 (< 2 foci/field), 2 (2–4 foci/field), or 3 (> 4 foci/field). Hepatocellular ballooning was scored as 0 (none), 1 (few/rare), or 2 (many/common). Mallory-Denk bodies were scored as 0 (absent), 1 (occasional), or 2 (several). Additionally, the fibrosis stage was evaluated using modified Mallory’s trichrome sections. It was graded on a five-point scale as follows: 0 (none), 1 (pericellular/perisinusoidal fibrosis), 2 (periportal fibrosis), 3 (focal bridging fibrosis), and 4 (cirrhosis) (Table S1). Two experienced pathologists independently scored the samples in a blinded manner. The sections were analyzed using a Leica DM2500 microscope with a built-in camera (Wetzlar, Germany). The image analyzer Leica Q win V.3 program was used for further analysis (Wetzlar, Germany).

### Investigating the regulatory networks of MASH-related genes using bioinformatics analysis

The public Gene Expression Omnibus (GEO) database (https://www.ncbi.nlm.nih.gov/gds/, accessed May 2022) was queried using specific keywords such as “MASH”, “Fatty liver”, “Fibrotic liver”, and “nonalcoholic steatohepatitis” to identify microarray datasets containing the most relevant genes for MASH. The selection criteria included: expression profiling tested by array, tissue samples collected from both MASH and normal samples, datasets used for analysis consisted of no less than five samples, ensuring sufficient statistical power, and these datasets contained comprehensive sample information. Based on these criteria, we selected three datasets: GSE140994, GSE93819, and GSE8253. Differentially expressed genes (DEGs) were identified using the GEO2R/R package limma with a cut-off point of *P*-value < 0.05 and |logFC| > 0.5 (Supplementary Tables S2 & S3). For enrichment analysis of MASH-related genes, the Gene Ontology (GO) analysis was performed using the GeneCards database (https://www.genecards.org/, accessed May 2022) (Fig S1). Six genes were selected: GPX4, LPCAT3, ACSL4, HGS, TSG101, and SNF8. Then, the STRING database was used for Protein-Protein Interaction analysis of the retrieved genes (https://string-db.org/, accessed May 2022) (Fig S2). To identify the epigenetic regulators of these DEGs, we first retrieved the miRNAs (rno-miR-329-5p, rno-miR-23a-5p, and rno-miR-27a-5p) interacting with the selected DEGs using the mirWalk database (http://mirwalk.umm.uni-heidelberg.de/, accessed May 2022) database and then identified the interacting lncRNAs (LINC00442 and CTBP1-AS2) using RNA22 (https://cm.jefferson.edu/rna22/, accessed May 2022) (Fig S3 & S4).

### RNA extraction and gene expression profiling in liver tissues

Total RNA was isolated from liver tissues using the miRNeasy kit (Qiagen, Hilden, Germany, cat. no. 74104) as per the manufacturer’s instructions. To ensure high-quality RNA, its concentration and purity were measured using the Qubit 3.0 Fluorometer (Invitrogen, Life Technologies, Malaysia) along with the Qubit TM ds DNA HS Assay Kit and Qubit TM RNA HS Assay Kit (Cat. no. Q32851, Q32852 respectively). Complementary DNA (cDNA) was synthesized from the purified RNA using a Rotor-Gene Thermal Cycler (Thermo Electron, Waltham, MA) and the QuantiTect Reverse Transcription Kit (Qiagen, Hilden, Germany, cat. no. 205311) following the kit protocol. RT-qPCR reactions were performed on a custom array 96-well plate designed with the pre-spotted Custom RT2 Profiler PCR Array (Qiagen, Hilden, Germany, cat. no. 330171) for the six target mRNAs. GAPDH and ACTB were used as reference genes. The QuantiTect SYBR Green PCR Kit (Qiagen, Hilden, Germany, cat. no. 204143) was used for detecting expression levels. For miRNAs, qPCR reactions were performed on a custom plate designed with the miRCURY LNA Custom miRNA PCR Panel (Qiagen, Hilden, Germany, cat. no. 339350 and cat. no. 339306) and the miRCURY LNA SYBR Green PCR Kit (Qiagen, Hilden, Germany, cat. no. 339345), using SNORD44 as an endogenous control. Additionally, the RT2 lncRNA qPCR Assay and QuantiNova LNA PCR Assay (Qiagen, Hilden, Germany, cat. no. 330701 & 249990) were used for quantifying lncRNAs with GAPDH as an endogenous control. The RT and PCR protocol was as follows: 95 °C for 2 min, followed by 45 cycles at 95 °C for 5 s and 60 °C for 10 s. Data were analyzed using the 2^−∆∆Cq^ method [32]. All cDNA samples were analyzed in duplicates using the 7500 Fast System from Applied Biosystems Real-time PCR. The specific primers and reagents were obtained from Qiagen, Germany, and are listed in Table S4.

### Measurement of rats’ liver function status and other covariates

Serum essential liver function parameters such as alanine transaminase (ALT), aspartate transaminase (AST), alkaline phosphatase (ALP), gamma-glutamyl transferase (GGT), total bilirubin (T. Bilirubin), and direct bilirubin (D. Bilirubin), in addition to Lipid analysis total cholesterol (TC), triglycerides (TG), high-density lipoprotein cholesterol (HDL-C), and low-density lipoprotein cholesterol (LDL-C), were determined using a Multifunctional Biochemistry Analyzer (AU680, Beckman Coulter Inc., Kraemer Blvd., Brea, CA 92821, USA). Furthermore, alpha-fetoprotein (AFP) levels were measured using an ELISA kit (Abcam, catalog number ab108838, Cambridge, MA, USA).

### Assessment of liver tissue cytokines and proteins

Interleukin-6 (IL-6) and transforming growth factor β1 (TGF-β1) levels in liver tissues were measured using sandwich ELISA kits (cat. nos. E0079r and E0124R, respectively; EIAab, Wuhan, China) following the manufacturer’s instructions. Additionally, TSG101, rat haptoglobin (Hpt), GPX4 (Sunredbio, cat. nos. 201-11-1828, 201-11-0358, and SRB-T-80533, respectively; Shanghai, China) were also quantified following the manufacturer’s instructions.

### Statistical analysis

Gene expression data were presented as fold change. Statistical analysis was carried out using SPSS 26 software. The data are expressed as mean ± SD, and significant differences were assessed using a 1-way analysis of variance followed by post hoc Tukey’s test. The normal distribution of the data was confirmed by the Shapiro-Wilk test. Categorical data were presented as percentages and compared using the chi-square test. A p-value of less than 0.05 was considered statistically significant.

## Machine learning model

### Building machine learning models

In this study, we utilized a combination of bioinformatics and machine learning techniques to identify key biomarkers for MASH and predict treatment outcomes. We examined the effects of four potential drugs—Febuxostat, Perindopril, Amlodipine, and Atorvastatin as well as the combination of Perindopril/Amlodipine by targeting biomarkers associated with MASH.

To comprehensively evaluate treatment effects and enhance predictive accuracy, key features were systematically compiled from the experimental results. These features included histopathological scores (e.g., NAS and fibrosis staging from Table S7), biochemical markers (e.g., liver enzymes, lipid profiles, bilirubin levels from Table 2), inflammatory and protein biomarkers (e.g., TGF-β1, IL-6, GPX4, TMAO from Table 3), and molecular expression profiles of ferroptosis/autophagy-related genes and non-coding RNAs (Table 1). By integrating these datasets, we generated a structured feature set to train and validate machine learning models aimed at predicting the degree of histological improvement following therapeutic interventions.

**Table 1:** Expression profiles of genes, miRNAs, and lncRNAs in the control, MASH, and treatment groups

	Control (*n* = 35)	MASH(*n* = 35)	Febuxostat-1.5 (*n* = 10)	Febuxostat-3(*n* = 10)	Febuxostat-6(*n* = 10)	Amlodipine(*n* = 10)	Perindopril(*n* = 10)	Amlodipine/Perindopril(*n* = 10)	Atorvastatin-20(*n* = 10)	*P*-value
GPX4	2.63 ± 0.83	0.002^a^	3.87 ± 0.43^b^	10.81 ± 1.68^ab^	38.38 ± 2.81 ^ab^	14.18 ± 1.62 ^ab^	16.21 ± 2.15 ^ab^	49.31 ± 5.92 ^ab^	0.88 ± 0.09	0.000
LPCAT3	0.97 ± 0.09	11.59 ± 1.27^a^	5.01 ± 0.65^ab^	3.07 ± 0.24^ab^	0.50 ± 0.05^b^	3.71 ± 0.48^ab^	3.09 ± 0.52^ab^	0.75 ± 0.11^b^	6.71 ± 0.87^ab^	0.000
ACSL4	1.39 ± 0.23	54.89 ± 5.81^a^	20.84 ± 1.65^ab^	10.39 ± 1.18^ab^	6.65 ± 1.08^ab^	11.87 ± 0.96^ab^	9.04 ± 0.79^ab^	1.16 ± 0.16^b^	27.93 ± 21^ab^	0.000
HGS	1.03 ± 0.06	74.38 ± 5.78^a^	54.81 ± 2.98^ab^	28.5 ± 2.09^ab^	7.62 ± 1.17^ab^	40.24 ± 3.09^ab^	33.65 ± 2.58^ab^	5.88 ± 0.45^ab^	62.49 ± 3.39^ab^	0.000
TSG101	1.06 ± 0.09	39.12 ± 2.79^a^	18.98 ± 2.02^ab^	8.18 ± 0.86^ab^	1.81 ± 0.38^b^	21.04 ± 1.55^ab^	16.13 ± 1.18^ab^	3.07 ± 0.23^ab^	25.43 ± 2.71^ab^	0.000
SNF8	1.18 ± 0.22	117.58 ± 11.13^a^	68.8 ± 7.82^ab^	22.41 ± 1.65^ab^	2.49 ± 0.23^b^	70 ± 6.69^ab^	64.95 ± 6.19^ab^	9.28 ± 0.89^ab^	92.2 ± 10.48^ab^	0.000
rno-miR-329-5p	2.94 ± 0.76	0.02 ± 0.03^a^	5.68 ± 0.83^ab^	14.18 ± 2.18^ab^	25.97 ± 2.77^ab^	9.44 ± 1.22^ab^	10.23 ± 1.91^ab^	24.17 ± 3.05^ab^	1.58 ± 2.1	0.000
rno-miR-27a-5p	0.95 ± 0.09	192.42 ± 12.92^a^	104.34 ± 4.47^ab^	57.05 ± 6.19^ab^	15.02 ± 2.45^ab^	91.11 ± 5.69^ab^	83.58 ± 5.21^ab^	12.48 ± 0.78^ab^	148.17 ± 6.35^ab^	0.000
rno-miR-23a-5p	1.04 ± 0.11	17.6 ± 2.16^a^	9.09 ± 0.88^ab^	2.75 ± 0.27^ab^	0.29 ± 0.05^b^	9.15 ± 1.09^ab^	8.36 ± 0.99^ab^	1.14 ± 0.13^b^	11.09 ± 1.08^ab^	0.000
LINC00442	1.03 ± 0.12	92.82 ± 6.59^a^	27.31 ± 2.57^ab^	14.94 ± 1.51^ab^	1.67 ± 0.19^b^	49.45 ± 3.41^ab^	38.54 ± 2.64^ab^	6.01 ± 0.41^ab^	40.41 ± 3.81^ab^	0.000
CTBP1-AS2	1.14 ± 0.18	80.38 ± 5.25^a^	38.38 ± 4.61^ab^	10.89 ± 1.16^ab^	1.13 ± 0.18^b^	41.69 ± 2.61^ab^	30.83 ± 1.94^ab^	5.22 ± 0.33^ab^	56.81 ± 6.83^ab^	0.000

The data are presented as mean ± SD. The statistically significant difference between groups was assessed with the ANOVA-Tukey post hoc test, where ‘a’ denotes statistical significance when compared to the control group, and ‘b; represents statistical significance when compared to the MASH group. A p-value of less than 0.05 is considered statistically significant

We categorized treatment improvement into four levels: ‘Not Improved’, ‘Light Improved’, ‘Medium Improved’, and ‘Significantly Improved’, based on NAS scores. Specifically, a score of 8 indicated ‘Not Improved’, scores of 6, 7, or 5 indicated ‘Light Improved’, scores of 4 or 3 indicated ‘Medium Improved’, and scores of 2, 1, or 0 indicated ’ Significantly Improved’. The analysis revealed the ratio for each improvement class, as shown in Figure S5.

Multi-classifier approaches were implemented using a Random Forest Classifier, Extra Trees Classifier, Logistic Regression, Linear Discriminant Analysis, and Light Gradient Boosting Machine. We split our data into 70% for training and 30% for testing. Our dataset included 140 mouse samples, divided into control, diseased (MASH-12 weeks), and treated (drug) groups (Table S5). We worked with three distinct feature types (Table S6) molecular, biochemical, and immunohistochemical markers.

**Fig. 1:**
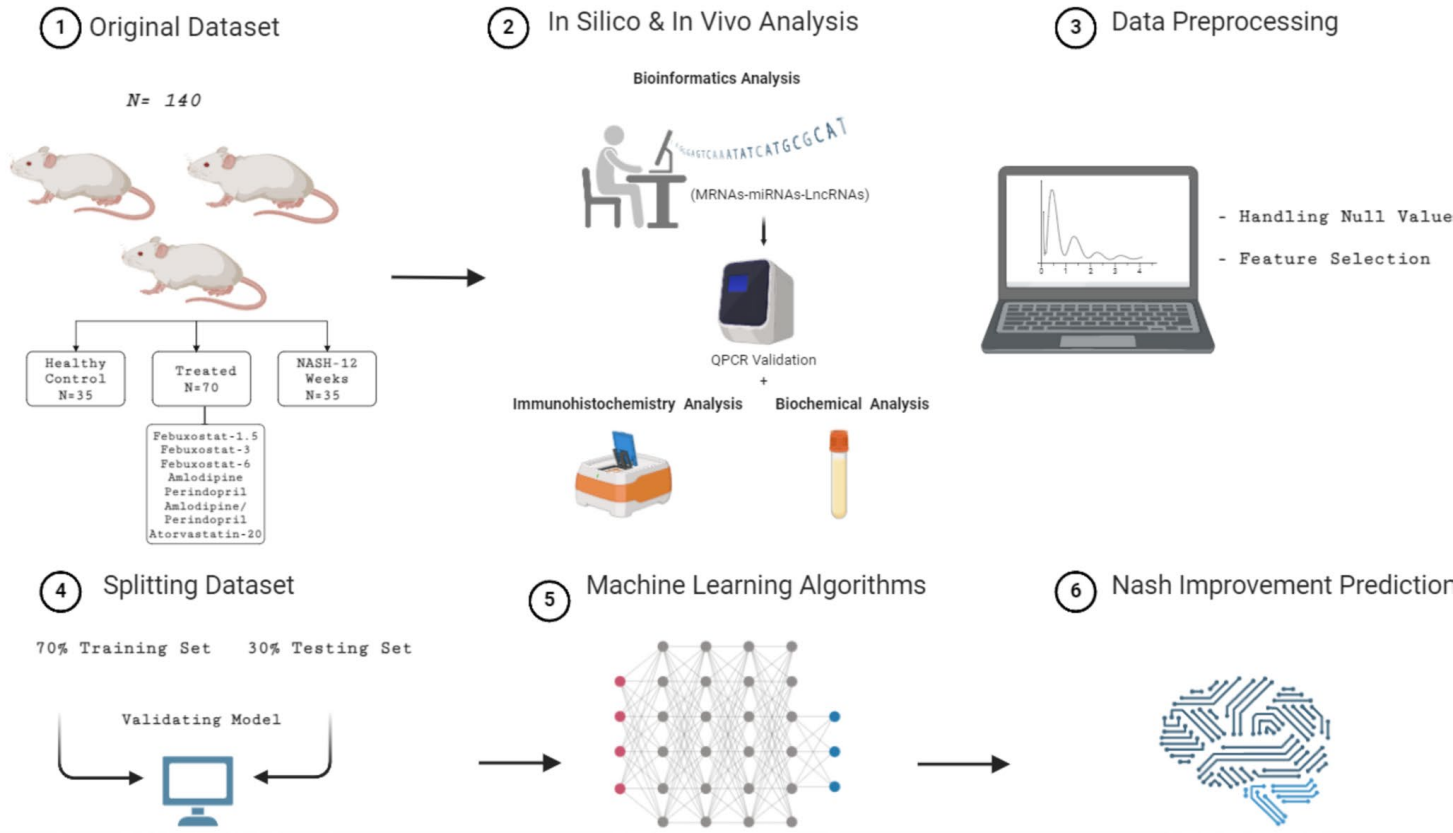
The overall workflow of the model

To thoroughly assess the predictive power of these features, we developed four models: one for each feature type and a final combined model that incorporated all features. This multi-faceted approach allowed us to evaluate the effectiveness of each drug and the role of various biomarkers in improving MASH treatment outcomes. The overall workflow of the model design is represented in Fig. 1.

### Data preparation

Preprocessing the data before applying it to classifiers is a crucial part of machine learning model development. Our process began with reading the original dataset, followed by dropping all features with null values to ensure data integrity. Noisy data was cleaned to enhance the dataset’s quality. We then created the target column, which categorized the NAS scores into improvement levels: ‘Not Improved’, ‘Light Improved’, ‘Medium Improved’, and ’ Significantly Improved’. These steps ensured that the dataset was prepared and ready for effective model training and evaluation.

### Correlation analysis

To investigate the relationships between various biomarkers we conducted a correlation analysis. The resulting correlation matrix displays the correlation coefficients for each pair of variables, with values ranging from − 1 to 1. Positive values indicate a direct relationship, whereas negative values indicate an inverse relationship. The magnitude of the correlation coefficient denotes the strength of the relationship, with values closer to 1 or −1 representing stronger correlations [33].

### Feature selection

Feature selection streamlines data by reducing its dimensionality and complexity, which enhances learning efficiency. By retaining only the relevant features, the model becomes faster and more accurate, while also improving its predictive performance by minimizing noise [34, 35].

### Feature importance

We utilized the Random Forest classifier to determine the importance of each feature in predicting treatment improvement for MASH. Feature importance provides insights into which variables contribute most significantly to the model’s predictions. By analyzing the feature importance scores, we were able to identify key biomarkers and clinical measurements that have the greatest impact on treatment outcomes. This step was crucial for understanding the underlying factors influencing MASH improvement and for guiding further feature selection and model refinement.

### Recursive feature elimination with cross-validation (RFECV) technique to select top features

To optimize our model and reduce complexity, we employed RFECV. This method iteratively removes the least important features and builds the model on the remaining features, using cross-validation to evaluate performance. RFECV helps in selecting the most relevant subset of features, enhancing the model’s predictive accuracy while avoiding overfitting. By applying RFECV, we identified a refined set of features that provided the best balance between model performance and simplicity, ensuring robust and interpretable results.

We employed a Random Forest Classifier as the estimator for RFECV, setting the step parameter to 1 to remove one feature at a time. A 5-fold cross-validation was used to validate the performance at each iteration, with accuracy as the primary scoring metric. This approach allowed us to systematically eliminate less important features, resulting in a refined and efficient model with a balanced trade-off between simplicity and predictive power.

### Cross validation approach

In k-fold cross-validation, the dataset is partitioned into k distinct and equally-sized subsets. The classifier is trained on the union of k-1 subsets and then evaluated on the remaining subset. This procedure is iterated k times, with each subset serving as the validation set exactly once. The overall error rate is estimated by averaging the error rates obtained from each iteration. Consequently, each data point is included in the validation set exactly once and appears in the training set k-1 times. Employing a larger value for k can help to minimize the variance of the error estimate, leading to a more robust evaluation of the model’s performance [36].

We applied 5-fold cross-validation to evaluate the performance of our machine learning models. Then used Stratified K-Fold cross-validation to ensure that each fold maintained the same class distribution as the overall dataset, thus minimizing potential biases and enhancing the reliability of our results. The dataset was split into five subsets, with each subset reflecting a representative distribution of the target variable classes. For each fold, we trained the model on four of the subsets and validated it on the remaining subset. This process was repeated five times, ensuring that each subset was used for validation exactly once. By implementing Stratified K-Fold cross-validation, we aimed to rigorously test our model on unseen data in each fold, thereby improving the accuracy and reproducibility of our results.

### Multi-class prediction of MASH disease improvement

In this study, for the prediction of MASH disease improvement, we utilized five distinct machine learning algorithms Random Forest Classifier, Extra Trees Classifier, Logistic Regression, Linear Discriminant Analysis, and Light Gradient Boosting Machine to develop predictive models. Models were trained and validated on four separate feature sets: molecular, biochemical, immunohistochemical, and a combined feature set. The models incorporated the most relevant features identified by RFECV. This approach enabled us to focus on the most impactful features, enhancing the accuracy and effectiveness of our predictions.

### Model evaluation metrics

To assess the performance of our predictive models, we employed several evaluation metrics. We used a 4 × 4 confusion matrix to analyze the classification results, which allowed us to evaluate the models’ ability to correctly classify the different levels of MASH disease improvement. Additionally, we calculated the Area Under the Curve (AUC) of the Receiver Operating Characteristic (ROC) curve to measure the overall discriminative power of each model. Key performance metrics including accuracy, precision, sensitivity, and F1 score were also computed.

### Packages selection and utilization

In this project, Python 3.12 was employed as the programming language for data processing. Several key packages were utilized to support various stages of data analysis, feature selection, and model development. ‘Pandas’ and ‘NumPy’ were essential for data manipulation and numerical operations, enabling efficient handling and preprocessing of the dataset. ‘Seaborn’ and ‘Matplotlib’ were employed for data visualization, facilitating the creation of informative plots and charts to explore and interpret the data. For machine learning tasks, ‘Scikit-learn’ aided in model development and evaluation. Additionally, ‘StratifiedKFold’ was used to ensure balanced folds during cross-validation. The ‘PyCaret’ library was also instrumental in streamlining the classification process, offering an automated environment for model training, evaluation, and comparison.

## Results

### Pathological assessment of hepatic biopsies

A total of 35 rats per group were assigned to the control and MASH groups, while all treatment groups consisted of 10 rats each. A comprehensive assessment of histological disease activity was conducted using the NAS, which includes grading of steatosis, ballooning, and lobular inflammation, alongside the fibrosis staging. The complete distribution of histological grades and stages is summarized in Table S7 and Figures S6 and S7. The control group displayed normal hepatic architecture, with central veins in the middle of lobules and portal tracts at the periphery. Hepatocytes had acidophilic cytoplasm and central vesicular nuclei, separated by narrow blood sinusoids (Figure S6a).

In the MASH group, disease severity was prominent, with 40% of animals scoring a NAS of 7, and 60% scoring 6, compared to the control group, where 100% of rats had a NAS score of 0. MASH rats showed severe steatosis (> 66%) in 57.14%, severe lobular inflammation (> 4 foci) in 68.5%, and extensive ballooning in 88.57%. Additionally, all MASH animals exhibited several Mallory-Denk bodies with dilated hepatic sinusoids and frequent intralobular mononuclear infiltration and hemosiderin/lipofuscin deposits (Figure S6b), and 34.29% had bridging fibrosis, with another 51.43% at stage 2 fibrosis.

Treatment groups showed varying degrees of histological improvement. The Febuxostat-6 group demonstrated the most pronounced improvement, with 80% of rats showing < 5% steatosis, 50% with no ballooning, minimal Mallory-Denk body presence in 70%, and NAS scores ranging from 0 to 2. Additionally, 80% had no fibrosis, and only 10% reached stage 1. The histopathology figures exhibited the most significant improvement in liver architecture, nearing normalcy. Most hepatocytes displayed acidophilic cytoplasm and vesicular nuclei, with hardly any vacuolated hepatocytes observed (Figure S6e) The Amlodipine/Perindopril combination was similarly effective, with 60% of animals scoring NAS 0–2, 40% showing no ballooning, and 60% displaying no fibrosis. These findings were reflected in substantial reductions in steatosis, ballooning, inflammation, and Mallory-Denk bodies compared to MASH and improved liver histology (Figure S6h).

In contrast, Febuxostat-1.5 and Atorvastatin-20 showed more moderate effects. Febuxostat-1.5 resulted in NAS scores of 4–5 in all animals, with 50% still showing ballooning in many hepatocytes, and only 30% exhibiting no fibrosis. Most hepatic lobules retained their structural integrity, with hepatocytes featuring central vesicular nuclei. Moreover, some hepatic areas exhibited vacuolated hepatocytes and varying degrees of fat accumulation, hepatocyte swelling, and Mallory-Denk bodies (Figure S6c). The Atorvastatin-20 group had 70% of animals with NAS scores of 5, 70% still with many ballooned hepatocytes, and 40% with bridging fibrosis (Figure S6i). Other groups showed partial improvements such as Febuxostat-3 displayed better-preserved liver architecture with fewer vacuolated hepatocytes, lowered NAS to 2–4, reduced steatosis to ≤ 33% in 90% of animals, and showed fibrosis in 50%, mostly stage 1 (Figure S6d). Amlodipine and Perindopril reduced inflammation and steatosis moderately, but fibrosis remained present in 70–80% of rats (Figure S6f-g) (Table S7).

In Sirius red-stained liver sections, the control group exhibited fine collagen fibers around the central vein, interspersed among hepatocytes, and portal tracts (Figure S7a). In the MASH group, there was a significant increase in collagen fibers, particularly in the periportal areas, with frequent bridging fibrosis and associations with macrovesicular steatosis (Figure S7b). The febuxostat-1 group showed focal increases in pericellular collagen fibers, while the febuxostat-3 group had fine collagen fibers in periportal areas and between hepatocytes (Figures S7c-d). The febuxostat-6 group displayed minimal collagen fibers, similar to the control group (Figure S7e). Amlodipine and perindopril groups had increased collagen fibers paracellularly and around portal tracts, with the combination group showing similar patterns around central veins and portal tracts (Figure S7f-h). The atorvastatin-treated group exhibited extensive collagen fibers in the periportal areas and between hepatocytes, associated with steatosis, similar to the MASH group (Figure S7i).

### Expression patterns of ferroptosis and autophagy markers in MASH pathogenesis

To investigate the role of ferroptosis and autophagy in MASH pathogenesis, we analyzed the expression profiles of key ferroptosis genes (GPX4, LPCAT3, and ACSL4) and autophagy genes (HGS, TSG101, SNF8), along with their epigenetic regulators, across control, MASH, and treated groups. The findings showed notable changes in the expression levels of these markers among the various treatment groups. Twelve weeks HSHFD-MASH group showed statistical significant increase in hepatic LPCAT3, ACSL4, HGS, TSG101, SNF8, rno-miR-329-5p, rno-miR-23a-5p, LINC00442, and CTBP1-AS2, while it reduced the GPX4, the antioxidant enzyme, and rno-miR-27a-5p. The highest dose of Febuxostat effectively decreased the levels of LPCAT3, TSG101, SNF8, rno-miR-23a-5p, LINC00442, and CTBP1-AS2 compared to the MASH group and surpassed the lower doses (Febuxostat-1.5&3 mg). Interestingly, the results demonstrate that the combination treatment of Amlodipine/Perindopril led to significantly higher modulation of biomarkers compared to single treatments, particularly in restoring ACSL4 levels, which are crucial for inducing ferroptosis, to the normal range seen in the control group. Meanwhile, Atorvastatin-20 displayed only minor alterations in biomarker expression relative to the control group (Table 1).

### Impact of therapeutic approaches on biochemical markers in MASH

To further characterize disease progression and treatment efficacy, key biochemical markers — including liver function enzymes (ALT, AST, ALP, GGT), bilirubin levels, serum album, AFP, and lipid profiles (total cholesterol, triglycerides, HDL-C, and LDL-C) — were analyzed across all groups (Table 2).

**Table 2:** Impact of pharmacological interventions on biochemical markers in the control, MASH, and treatment Groups

	Control (*n* = 35)	MASH(*n* = 35)	Febuxostat-1.5 (*n* = 10)	Febuxostat-3(*n* = 10)	Febuxostat-6(*n* = 10)	Amlodipine(*n* = 10)	Perindopril(*n* = 10)	Amlodipine/Perindopril(*n* = 10)	Atorvastatin-20(*n* = 10)	*P*-value
Body Weight (gm)	281.20 ± 35.89	351.86 ± 52.92	328.20 ± 32.84	330 ± 56.99	336.50 ± 57.28	281.40 ± 35.32	358.22 ± 49.02	350.60 ± 24.60	286.20 ± 26.67	0.013
ALT (IU/L)	41.09 ± 6.33	137.43 ± 7.57^ab^	95.6 ± 7.88^ab^	68 ± 6.07^ab^	46.4 ± 7.2^b^	71.7 ± 10.37^ab^	61.90 ± 9.09^ab^	42.70 ± 5.36^b^	106.20 ± 8.79^ab^	0.000
AST (IU/L)	38.89 ± 4.46	102.54 ± 7.38^a^	81.80 ± 5.75^ab^	65.60 ± 4.43^ab^	42.50 ± 4.03^b^	68.20 ± 7.32^ab^	58.9 ± 6.24^ab^	40.90 ± 4.56^b^	90.90 ± 6.26^ab^	0.000
ALP (IU/L)	43.29 ± 6.89	136.77 ± 7.60^ab^	105.30 ± 5.87^ab^	61.80 ± 7.19^ab^	47.81 ± 3.68^b^	75.50 ± 11.91^ab^	65.20 ± 10.12^ab^	45.4 ± 7.32^b^	116.90 ± 6.51^ab^	0.000
GGT (IU/L)	18.56 ± 1.93	84.26 ± 10.09^a^	64.20 ± 4.92^ab^	35 ± 3.86^ab^	19.55 ± 1.22^b^	33.19 ± 3.53^ab^	28.63 ± 3.06^ab^	19.31 ± 3.19^b^	71.30 ± 5.59^ab^	0.000
T.Bilirubin(mg%)	0.31 ± 0.08	1.74 ± 0.25^a^	1.22 ± 0.24^ab^	0.61 ± 0.09^ab^	0.44 ± 0.05^b^	0.58 ± 0.15^ab^	0.51 ± 0.13^ab^	0.35 ± 0.09^b^	1.36 ± 0.19^ab^	0.000
D.Bilirubin(mg%)	0.16 ± 0.03	0.75 ± 0.09^a^	0.54 ± 0.14^ab^	0.28 ± 0.03^ab^	0.18 ± 0.03^b^	0.29 ± 0.05^ab^	0.26 ± 0.04^ab^	0.18 ± 0.03^b^	0.63 ± 0.16^ab^	0.000
AFP (ng/mL)	22.83 ± 3.69	612.49 ± 102.16^a^	349.12 ± 27.72^ab^	157.94 ± 19.27^ab^	78.32 ± 6.32^b^	41.60 ± 6.55^b^	35.9 ± 5.57^b^	27.91 ± 3.48^b^	385.08 ± 30.57^ab^	0.000
Albumin(g/dL)	4.08 ± 0.37	1.31 ± 0.27^a^	2.26 ± 0.42^ab^	2.86 ± 0.19^ab^	3.17 ± 0.35^ab^	3.02 ± 0.28^ab^	3.22 ± 0.30^ab^	3.77 ± 0.35^b^	2.12 ± 0.39^ab^	0.000
TC(mg%)	93.95 ± 13.39	154.88 ± 14.77^a^	114.88 ± 8.56^ab^	91.54 ± 4.95^b^	92.84 ± 4.19^b^	132.99 ± 18.69^ab^	122.89 ± 17.27^ab^	105.48 ± 14.82^b^	124.29 ± 9.37^ab^	0.000
TG(mg%)	60.59 ± 8.99	207.61 ± 12.89^a^	146.11 ± 11.93^ab^	113.32 ± 6.05^ab^	83.31 ± 8.95^ab^	84.88 ± 12.67^ab^	78.44 ± 11.71^ab^	67.32 ± 10.05^b^	157.91 ± 13.03^ab^	0.000
HDL-C(mg%)	60.54 ± 11.54	29.75 ± 5.03^a^	38.21 ± 4.32^a^	45.02 ± 5.49 ^ab^	45.69 ± 3.89 ^ab^	42.52 ± 6.57 ^ab^	49.63 ± 11.75 ^ab^	48.18 ± 6.71 ^ab^	41.40 ± 4.62 ^ab^	0.000
LDL-C(mg%)	17.29 ± 2.29	82.61 ± 5.27^a^	66.87 ± 5.03^ab^	39.49 ± 5.39^ab^	20.54 ± 2.03^b^	31.72 ± 4.47^ab^	27.36 ± 3.85^ab^	19.36 ± 2.61^b^	72.20 ± 5.61^ab^	0.000

The data are presented as mean ± SD. The statistically significant difference between groups was assessed with the ANOVA-Tukey post hoc test, where ‘a’ denotes statistical significance when compared to the control group, and ‘b; represents statistical significance when compared to the MASH group. A *P*-value of less than 0.05 is considered statistically significant

Compared to the control group, MASH animals exhibited substantial elevations in liver enzymes, total and direct bilirubin, and AFP, alongside significant reductions in serum albumin levels. These findings were indicative of pronounced hepatocellular injury, impaired liver synthetic capacity, and systemic metabolic disturbances associated with MASH progression. Treatment with Febuxostat-6 and the Amlodipine/Perindopril combination resulted in the most pronounced biochemical improvements. Both groups showed enhancement of liver enzymes and bilirubin levels, restoration of serum albumin concentrations, and marked reductions in AFP levels. Improvements in lipid profiles are characterized by reductions in total cholesterol, triglycerides, and LDL-C levels, as well as increases in HDL-C levels.

Moderate biochemical amelioration was observed in the Febuxostat-3, Amlodipine, and Perindopril monotherapy groups, which demonstrated partial normalization of liver function markers and lipid parameters. However, Febuxostat-1.5 and Atorvastatin-20 treatments were associated with only modest improvements, with persistently elevated liver enzymes and AFP levels, and less effective restoration of albumin and lipid profiles compared to higher-dose or combination therapies. Although numerical differences in body weight were observed across groups, no significant differences were found between the control, MASH, and treatment groups at the study endpoint.

### Inflammatory and protein biomarker modulation in MASH treatment groups

The hepatic levels of Haptoglobin (Hpt), IL-6, TMAO, and TGF-β1 were utilized as indicators of inflammation and fibrogenesis, alongside GPX4 and TSG101 proteins, across various treatment groups, showing significant differences. Haptoglobin levels significantly decreased in the MASH group (632.77 ± 92.72 µg/g) compared to the control (1747.06 ± 291.54 µg/g). Among the treatments, Febuxostat at 6 mg and the Amlodipine/Perindopril combination exhibited the most remarkable improvements, with haptoglobin levels reaching 1626.48 ± 176.97 µg/g and 1820.33 ± 144.09 µg/g, respectively. Elevated TMAO levels in the MASH group (1474.05 ± 97.98 ng/mg) were notably reduced by Febuxostat-6 (451.12 ± 34.31 ng/mg) and Amlodipine/Perindopril (419.54 ± 31.91 ng/mg). Reduced GPX4 levels in the MASH group (292.9 ± 34.52 ng/mg) were significantly restored by Febuxostat-6 (1485.59 ± 191.14 ng/mg) and the Amlodipine/Perindopril combination (1604.44 ± 206.43 ng/mg). Similarly, TSG101 and IL-6, which were elevated in MASH, showed considerable reductions with Febuxostat-6 and Amlodipine/Perindopril treatments. Overall, the Amlodipine and Perindopril combination therapy demonstrated superior efficacy in modulating these biomarkers compared to other treatments (Table 3).

**Table 3:** Impact of treatments on key inflammatory and protein biomarkers in control, MASH, and treated groups

	Control	MASH	Febuxostat-1.5	Febuxostat-3	Febuxostat-6	Amlodipine	Perindopril	Amlodipine/Perindopril	Atorvastatin-20	*P*-value
Hpt (ug/gm)	1747.06 ± 291.54	632.77 ± 92.72^a^	872.27 ± 130.41^ab^	1126.18 ± 135.51^ab^	1626.48 ± 176.97^b^	1111.28 ± 88.56^ab^	1308.89 ± 139.16^ab^	1820.33 ± 144.09^b^	722.71 ± 94.61^a^	0.000
TMAO (ng/mg)	372.24 ± 24.67	1474.05 ± 97.98^a^	902.87 ± 62.62^ab^	643.34 ± 44.62^ab^	451.12 ± 34.31^ab^	694.82 ± 48.19^ab^	598.28 ± 41.49^ab^	419.54 ± 31.91^b^	975.09 ± 67.63^ab^	0.000
GPX4 (ng/mg)	1534.82 ± 180.87	292.91 ± 34.52^a^	427.87 ± 56.57^a^	798.69 ± 105.59^ab^	1485.59 ± 191.14^b^	766.75 ± 101.37^ab^	894.54 ± 118.27^ab^	1604.44 ± 206.43^b^	389.36 ± 51.48^ab^	0.000
TSG101 (ng/mg)	2914.49± 531.56	10965.78± 979.28^a^	8107.03± 724.62^ab^	5600.74± 364.33^ab^	3446.79± 287.99^b^	5375.22± 974.91^ab^	4635.77± 840.82^ab^	3270.63± 593.19^b^	8917.73± 797.09^ab^	0.000
IL-6 (ρg/g)	1040.89± 189.84	3916.35± 349.74^a^	2895.37± 258.79^ab^	2000.26± 130.12^ab^	1231± 102.86^b^	1919.72± 348.18^ab^	1655.63± 300.28^ab^	1168.08± 211.86^b^	3184.88± 284.67^ab^	0.000
TGFβ1 (ρg/g)	1428.69± 239.39	5499.82± 558.47^a^	3515.02± 349.53^ab^	2522.04± 173.76^ab^	2017.51± 81.94^ab^	2630.73± 439.67^ab^	2268.83± 379.19^ab^	1600.71± 267.52^b^	3866.53± 384.48^ab^	0.000

The data are presented as mean ± SD. The statistically significant difference between groups was assessed with the ANOVA-Tukey post hoc test, where ‘a’ denotes statistical significance when compared to the control group, and ‘b; represents statistical significance when compared to the MASH group. A *P*-value of less than 0.05 is considered statistically significant

### Feature correlation and importance analysis

**Fig. 2:**
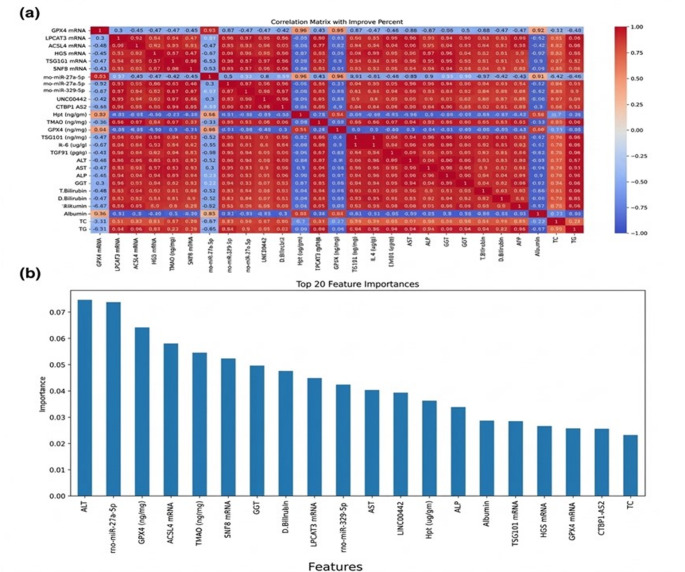
Correlation matrix and feature importance analysis. (**a**) Correlation matrix representing the pairwise relationships between features across the MASH dataset. (**b**) Bar chart illustrating the top 20 features ranked by their importance scores

The correlation matrix, illustrated in Fig. 2a, revealed the correlation levels between feature pairs across the entire dataset, offering insights into the relationships among the features. Each cell in the matrix displayed the corresponding correlation coefficient. To assess the contribution of individual features to the model’s performance. The importance of each feature was calculated based on its impact on the model’s decision-making process. Figure 2b illustrates the top 20 features ranked by their importance, as determined by the classifier. This analysis helps in identifying the most relevant features, guiding subsequent feature selection and model refinement efforts.

### Key features selected by RFECV

**Fig. 3:**
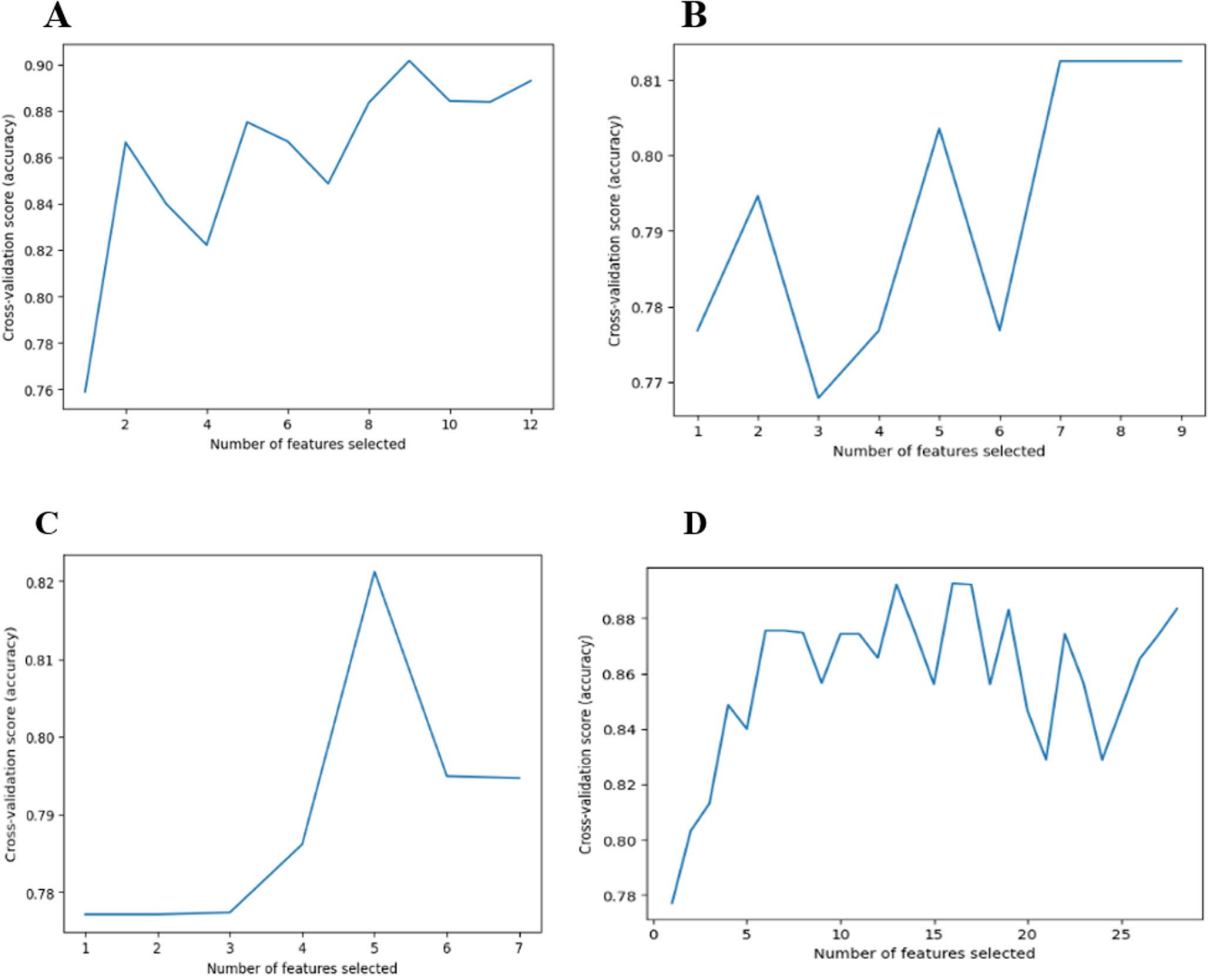
Feature Selection Performance Using RFECV. (**A**) Molecular, (**B**) Biochemical, (**C**) Immunohistochemical, (**D**) Combined set

Feature selection using RFECV identified the most critical predictors for the improvement levels in MASH treatment. The analysis of RFECV and the accuracy on the test set for each feature group are shown in Fig. 3 out of the original features, the RFECV method selected 9 out of 11 features for the molecular model, 11 out of 12 features for the biochemical, 5 out of 6 features for the immunohistochemistry, and 16 out of 29 features for the combined model these selections were done while keeping the same prediction accuracy level. The included and excluded features with accuracy scores are shown in Table S8. These findings underscore the relevance of these selected biomarkers in predicting the treatment outcomes for MASH.

### Evaluation of ML models in predicting MASH improvement

**Fig. 4:**
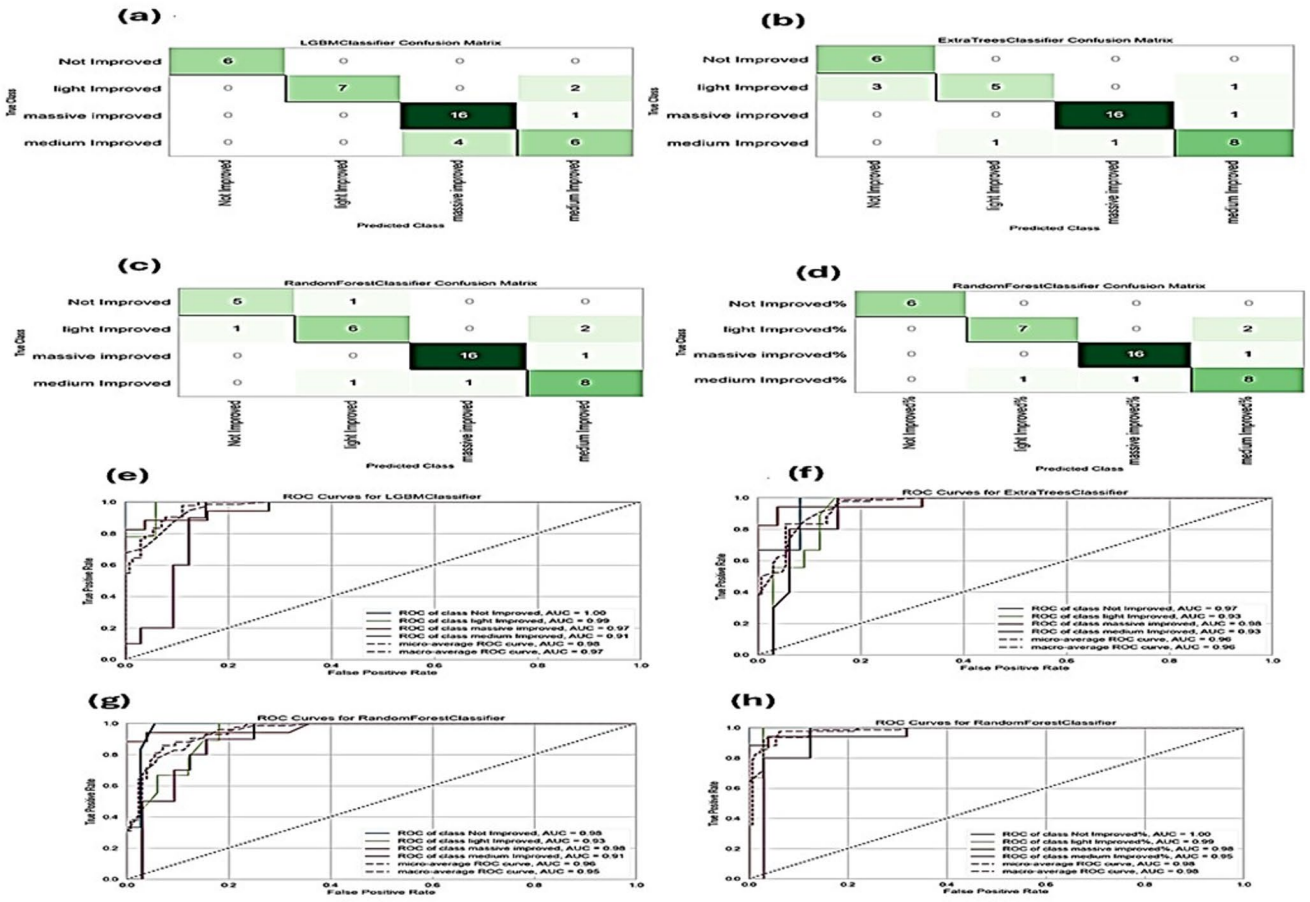
Confusion matrices and ROC curves for the combined model. (**a**-**d**) Confusion Matrix for top classifier prediction for each feature group. (**a**) Molecular, (**b**) Biochemical, (**c**) Immunohistochemical, (**d**) Combined. (**e**-**h**) Roc curve for the top-performing classifier for each feature set. (**e**) Molecular, (**f**) Biochemical, (**g**) Immunohistochemical, (**h**) Combined

The performance of each model is shown in Table 4. The confusion matrix presented in Fig. 4 (a-d) illustrates the accuracy of the predictions for the classes “Not Improved,” “Light Improved,” " Significantly Improved,” and “Medium Improved” on the test set for the combined model while the Roc curve displays how accurate the prediction models were Fig. 4 (e-h).

**Table 4:** Comparison of the performance of the different machine-learning algorithms for each model

Model (Molecular)	Accuracy	AUC	Recall	Precision	F1-Score
Random Forest	0.8570	0.9634	0.8570	0.8846	0.8583
Extra Trees Classifier	0.8772	0.9645	0.8772	0.9050	0.8809
Logistic Regression	0.8374	0.9645	0.8374	0.8373	0.8344
Linear Discriminant Analysis	0.8479	0.9547	0.8479	0.8581	0.8488
Light Gradient Boosting Machine	**0.8778**	**0.9570**	**0.8778**	**0.8816**	**0.8770**
Model (Biochemical)	**Accuracy**	**AUC**	**Recall**	**Precision**	**F1-Score**
Random Forest	0.8261	0.9655	0.8261	0.8358	0.8250
Extra Trees Classifier	**0.8264**	**0.9646**	**0.8264**	**0.8429**	**0.8261**
Logistic Regression	0.7961	0.9541	0.7961	0.8133	0.7923
Linear Discriminant Analysis	0.7658	0.9372	0.7658	0.7573	0.7529
Light Gradient Boosting Machine	0.7655	0.9456	0.7655	0.7725	0.7620
Model (Immunohistochemical)	Accuracy	AUC	Recall	Precision	F1-Score
Random Forest	**0.8475**	**0.9461**	**0.8475**	**0.8633**	**0.8473**
Extra Trees Classifier	0.7762	0.9490	0.7762	0.7855	0.7753
Logistic Regression	0.7964	0.9529	0.7964	0.8124	0.7925
Linear Discriminant Analysis	0.7762	0.9570	0.7762	0.7995	0.7649
Light Gradient Boosting Machine	0.7759	0.9408	0.7759	0.7790	0.7699
Model (Combined)	Accuracy	AUC	Recall	Precision	F1-Score
Random Forest	**0.8874**	**0.9745**	**0.8874**	**0.9084**	**0.8875**
Extra Trees Classifier	0.8874	0.9690	0.8874	0.9079	0.8887
Logistic Regression	0.8779	0.9719	0.8779	0.8928	0.8782
Linear Discriminant Analysis	0.8774	0.9580	0.8774	0.8909	0.8784
Light Gradient Boosting Machine	0.8768	0.9594	0.8768	0.8874	0.8768

## Discussion

MASH, a serious and inflammatory form of MASLD, can progress to cirrhosis and hepatocellular carcinoma [37, 38]. Despite the urgent need for effective treatments to halt or reverse MASH, no approved drugs are currently available, making its development particularly challenging [39]. Programmed cell death pathways — including ferroptosis, autophagy, apoptosis, and pyroptosis — play central roles in MASH pathogenesis, influencing liver inflammation, fibrosis, and carcinogenic potential [40, 41]. The key molecules involved in these processes are central targets for developing therapeutic strategies to address MASLD/MASH.

Our findings indicate that rats subjected to the HSHFD diet for 12 W exhibited elevated NAS scores, liver inflammation, and fibrosis as revealed by histological analysis. This was accompanied by elevated serum levels of liver function markers such as AST, ALT, ALP, GGT, total bilirubin, and direct bilirubin. The expression of key autophagic pathway genes (GPX4, LPCAT3, and ACSL4) was altered compared to control cells, suggesting that autophagy impairments may link hepatic steatosis to liver injury in MASH. Additionally, the expression of ferroptosis genes (HGS, TSG101, and SNF8) was also disrupted in the MASH model. The pathogenesis of MASH is closely associated with chronic lipid accumulation in the liver, evidenced by a significant increase in lipid profile markers and a decrease in HDL-C. To evaluate the impact of MASH on liver inflammation, we measured hepatic inflammatory markers. MASH was associated with increased hepatic levels of TGFβ, IL-6, and TMAO, while Hpt levels were decreased.

Furthermore, we measured the expression levels of miRNAs (rno-miR-27a-5p, rno-miR-329-5p, and rno-miR-23a-5p), and LncRNAs (LINC00442 and CTBP1-AS2). Results indicated that rno-miR-329-5p, rno-miR-23a-5p −5p, LINC00442, and CTBP1-AS2 were upregulated in the MASH group, while rno-miR-27a-5p was downregulated compared to the control group. Febuxostat-6 adjusted the levels of rno-miR-23a-5p, LINC00442, and CTBP1-AS2 back to normal. Thus, LINC00442 and CTBP1-AS2 may influence MASH by interacting with miRNAs rno-miR-27a-5p, rno-miR-329-5p, and rno-miR-23a-5p, and regulating gene expression patterns related to autophagy and ferroptosis.

Ferroptosis, an iron-dependent form of non-apoptotic cell death driven by membrane lipid peroxidation, plays a significant role in the progression of various liver diseases, including MASH [42–44]. Polyunsaturated fatty acids (PUFAs) are the primary substrates for lipid peroxidation during ferroptosis [45]. The ACSL4 is a key player, as it preferentially catalyzes the esterification of PUFAs, leading to the accumulation of oxidized phospholipids that trigger susceptibility to ferroptosis in MASH [46]. Additionally, LPCAT3 is essential for the reacylation of phospholipids, facilitating the integration of unsaturated fatty acids catalyzed by ACSL4 [47]. By incorporating PUFAs into cellular phospholipids, ACSL4 and LPCAT3 play a central role in mediating ferroptosis [48]. HGS is integral to the Endosomal Sorting Complex Required for Transport (ESCRT), essential for protein sorting and degradation. HGS deficiency disrupts the lysosomal balance in cardiomyocytes, leading to lysosomal storage disorders with abnormal autophagosome accumulation and protein aggregation [49, 50]. In mammalian cells, loss of HGS increases autophagy markers, indicating its role in autophagosome maturation and lysosomal homeostasis [51]. Tsg101 plays a crucial role in regulating intracellular processes such as transcription, cell growth, differentiation, cell signaling, and autophagy-mediated protein clearance. It is a component of the ESCRT-I complex, involved in the sorting of ubiquitinated proteins into endosomes [52].

Obesity has recently been characterized as a state of low-grade chronic inflammation, which is linked to the development of MASLD [53, 54]. Inflammatory markers typically associated with obesity include elevated levels of pro-inflammatory cytokines such as TGF-β1, IL-6, and haptoglobin, a marker of the liver’s acute phase response to inflammation [55]. TMAO, a metabolite produced by gut bacteria, has emerged as a potential risk factor for MASLD [56]. Elevated TMAO levels in the plasma contribute to MAFLD by influencing bile acid metabolism, the unfolded protein response, and oxidative stress [57]. Additionally, consuming carnitine, a precursor of TMAO, leads to liver inflammation by increasing hepatic levels of IL-1, IL-6, TNF-α, and TNF-β [58]. Research indicates that TMAO exerts pro-oxidative, pro-inflammatory, and pro-fibrotic effects by activating key inflammatory pathways such as TGF-βRI/Smad2, PERK/Akt/mTOR, and the NLRP3 inflammasome [59].

Febuxostat, commonly used to lower uric acid levels, has shown promising effects in alleviating hepatic inflammation, and cell damage, and restoring organ functions [60, 61]. Mechanistically, Febuxostat boosts GPX4 expression, activates the Keap1/Nrf2 pathway, and inhibits the TLR4/NF-κB p65 pathway [62]. Febuxostat exerts significant antioxidant and anti-inflammatory effects in various experimental models, reducing the production of inflammatory cytokines such as IL-1β and TNF-α [63, 64]. Our results align with these findings, showing that the highest dose of Febuxostat provides a pronounced hepatoprotective effect. It significantly reduces inflammatory markers, IL-6 levels, NAS scores, hepatic inflammation, and fibrosis stages. Moreover, Febuxostat decreases lipid markers like TC and LDL-C and improves overall liver function. We also observed modulation of ferroptosis and autophagy markers, with increased GPX4 expression and reduced levels of LPCAT3, TSG101, and SNF8 compared to the MASH model.

Amlodipine is commonly used to manage high blood pressure.[65]. Recent studies have shown that amlodipine also offers liver protection and anti-inflammatory benefits in mice, and it has been found to improve lipid profiles and glucose metabolism in both humans and rats [66, 67]. Our results indicated that amlodipine markedly lowered liver enzyme levels, enhanced lipid profiles, and reduced ferroptosis and autophagy markers. It also led to a decrease in NAS scores, fibrosis stages, and liver inflammation.

Studies have shown that angiotensin-converting enzyme inhibitors (ACEIs) like perindopril can protect against liver damage and reduce fibrosis in experimental models by modulating levels of TGF-β1 and Smad proteins [68, 69]. Moreover, our findings demonstrated that the administration of perindopril significantly decreased the levels of liver enzymes, improved lipid profiles, and decreased markers associated with ferroptosis and autophagy compared to the MASH-induced model. Additionally, perindopril resulted in a reduction in NAS scores, stages of fibrosis, and inflammation in the liver.

The Perindopril/amlodipine combination stands out as a particularly effective treatment for hypertension [70]. However, its impact on MASH treatment had not been investigated prior to this study. We discovered that the combination significantly modulates biomarkers related to ferroptosis and autophagy, notably reducing LPCAT3, ACSL4, and rno-miR-23a-5p to levels seen in the control group. Additionally, this combination more effectively lowered hepatic enzyme levels and improved lipid profiles compared to monotherapy. It also resulted in reduced NAS scores, fibrosis stages, and liver inflammation.

Atorvastatin, an HMG-CoA reductase inhibitor, reduces hepatic cholesterol synthesis by blocking the conversion of HMG-CoA to mevalonate, leading to enhanced LDL receptor expression in hepatocytes and increased clearance of LDL cholesterol from the bloodstream [71]. Additionally, atorvastatin possesses antioxidant, anti-apoptotic, and anti-inflammatory properties [72]. However, studies have shown that long-term use and high doses of atorvastatin, a subset of statin drugs, can lead to liver complications, increased cell apoptosis, and even mortality [73]. Treatment with Atorvastatin-20 produced partial improvements in several biochemical and molecular markers; however, these effects were not uniformly distributed across all biomarkers. While modest reductions were observed in parameters such as ALT, AST, and some inflammatory markers, key biomarkers like TSG101, TMAO, and markers of the ferroptosis pathways remained elevated compared to control levels. These findings suggest that Atorvastatin-20 exerted only limited therapeutic efficacy in this MASH model, with incomplete resolution of the underlying molecular disturbances.

Machine learning has transformed the medical field, becoming essential in early disease diagnosis and prediction. It enhances medical specialists’ decisions by enabling swift and precise disease diagnosis [74]. In this study, we integrated bioinformatics and machine learning techniques to identify biomarkers for MASH and predict treatment responses. These findings were validated histologically using the NAS score. Our analysis included three feature types molecular, biochemical, and immunohistochemical examined individually and in a combined model. This comprehensive approach allowed for a thorough assessment of feature importance and model performance in predicting MASH treatment outcomes.

To enhance model performance, we employed RFECV for feature selection. This process identified key features for the combined model, including the molecular features (LPCAT3, HGS, TSG101, SNF8, rno-miR-27a-5p, rno-miR-329-5p, LncRNA-CTBP1-AS2), Biochemical features (ALT, AST, ALP, GGT, D.Bilirubin, and Albumin), and immunohistochemistry features (TMAO, GPX4, TGFβ1). All models were evaluated using 5-fold cross-validation to ensure robustness. This comprehensive approach enabled a thorough assessment of feature importance and model performance in predicting MASH treatment outcomes.

Light Gradient Boosting achieved the highest accuracy for molecular features, while Random Forest and Extra Trees performed best for biochemical and immunohistochemical features. Notably, combining all features yielded the strongest overall performance, with Random Forest demonstrating superior predictive ability, supported by consistently high AUC values across models.

The Random Forest classifier demonstrated strong predictive performance across all improvement classes in the combined model. The ROC curve analysis showed excellent AUC values: 1.00 for Not Improved, 0.99 for Light Improved, 0.95 for Medium Improved, and 0.98 for Significantly Improved, with both micro-average and macro-average AUCs at 0.98. Confusion matrix analysis further confirmed the model’s accuracy, with all six Not Improved samples and sixteen out of seventeen Significantly Improved samples correctly classified. In the Light Improved group, seven of nine samples were accurately classified, with two misclassified as Medium Improved, while in the Medium Improved group, eight of ten samples were correctly classified, demonstrating the model’s strong ability to differentiate between varying levels of treatment response. These results highlight the efficacy of the Random Forest classifier in predicting MASH improvement levels, with high accuracy and AUC values. Also, the overall performance metrics underscore the reliability of the selected feature sets in contributing to the predictive power of the model.

Recent advances in machine learning applications for MASH diagnosis have highlighted the potential of integrating clinical, biochemical, and omics data for non-invasive disease staging. Stefanakis et al. developed three novel predictive models using a categorical gradient boosting machine (CatBoost) pipeline, validated through a classic 4:1 split and secondary independent cohort analysis. Their models, based on a limited set of clinical and metabolomic features, achieved high diagnostic accuracy (AUROC ~ 0.89) for detecting significant fibrosis (F2–F3) and outperformed twenty-three existing biomarker-, imaging-, and algorithm-based non-invasive tests, using both standard and reoptimized cutoffs. Their work emphasized the potential for lightweight, interpretable models to reliably predict MASH progression in clinical settings [75]. Similarly, Sanyal et al. applied large-scale serum proteomics combined with multivariate machine learning to define distinct protein signatures corresponding to steatosis, inflammation, ballooning, and fibrosis. They proposed a non-invasive “liquid biopsy” approach for staging MASLD/MASH, providing a promising alternative to traditional histological assessment [76].

Ghandian et al. used electronic health record data from 700 volunteers to detect progression from NAFL to MASH. After selecting key features, they applied an extreme gradient boosting classifier (XGBoost), which achieved an AUC of 0.79 for NAFL to MASH progression and 0.87 for fibrosis detection [77]. Naderi et al. employed various supervised learning algorithms—including logistic regression, support vector machine SVM, adaptive boosting AdaBoost, LightGBM, Random Forest, and XGBoost—to predict MASH using the NAS score. They incorporated extensive hyperparameter tuning, repeated leave-one-out cross-validation, and multiple feature selection methods (SFS, MI, ANOVA, chi-squared). Their best model, Random Forest, reached an accuracy of 81.32% ± 6.43% [78].

Wu et al. used clinical data from 577 patients to predict fatty liver disease with machine learning. Key selected biomarkers included triglycerides, HDL, glucose, age, sex, systolic and diastolic blood pressure, ALT, and AST, they tested several algorithms such as naive Bayes, neural networks, logistic regression, and RF. RF performed best in terms of accuracy and AUC [79].

The strengths of this study lie in its comprehensive integration of mRNAs, miRNAs, and LncRNAs, which are closely linked to MASH pathogenesis, alongside traditional biochemical and inflammatory markers, providing a robust set of targets for MASH prognosis. Additionally, comparing the outcomes to liver biopsy histology enhances the validity of the findings. However, the study has certain limitations. The sample size was insufficient, and although animal experiments supported the bioinformatics analysis, they did not fully capture the complexity of MASH, necessitating further validation with human clinical samples. Additionally, our models are limited to predicting the direct effects of specific drug targets, without evaluating potential unintended off-target effects. Lastly, reliance on RNA sequencing data from existing databases introduces potential biases in the analysis.

## Conclusion

In conclusion, our study successfully utilized bioinformatic analyses to identify significant drug targets in MASH associated with ferroptosis and autophagy pathways. By integrating data on biochemical, inflammatory, and pathological markers, we built ML models that could predict MASH improvement based on NAS scores. The identified targets, validated through quantitative real-time PCR, Eliza, and conventional biochemical analysis, demonstrate the potential for new therapeutic interventions in treating MASH. Our findings highlight the efficacy of using advanced computational methods to unravel complex disease mechanisms and pave the way for precision medicine approaches in liver disease treatment. The high accuracy and AUC of our models underscore the robustness of this integrative strategy, offering a promising direction for future research and drug development.

## Supplementary information

Below is the link to the electronic supplementary material.

Supplementary Material 1 (XLSX 6.02 MB)

Supplementary Material 2 (DOCX 12.8 MB)

## Data Availability

No datasets were generated or analysed during the current study.

## Abbreviations

NAFLD: Non-alcoholic fatty liver disease
MAFLD: Metabolic-associated fatty liver disease
MASLD: metabolic dysfunction-associated steatotic liver disease
T2D: type 2 diabetes
MASH: Metabolic dysfunction asssociated steatohepatitis
HCC: hepatocellular carcinoma
HSCs: hepatic stellate cells
PUFAs: polyunsaturated fatty acids
ACSL4: acyl-CoA synthetase long-chain family member 4
UA: uric acid
ROS: reactive oxygen species
XO: xanthine oxidase
RAS: renin-angiotensin system
AT-II: Angiotensin-II
ACE: angiotensin-converting enzyme
ARBs: AT1 receptor blockers
miRNAs: microRNAs
LncRNAs: long non-coding RNAs
ML: Machine Learning
HSHFD: high sucrose and high-fat diet
HE: Hematoxylin-eosin
NAS: MASH activity score
MASH CRN: MASH Clinical Research Network
GEO: Gene Expression Omnibus
DEGs: Differentially expressed genes
GO: Gene Ontology
cDNA: Complementary DNA
ALT: alanine transaminase
AST: aspartate transaminase
ALP: alkaline phosphatase
GGT: gamma-glutamyl transferase
T. Bilirubin: total bilirubin
D. Bilirubin: direct bilirubin
TC: total cholesterol
TG: Triglycerides
HDL-C: high-density lipoprotein cholesterol
LDL-C: low-density lipoprotein cholesterol
AFP: alpha-fetoprotein
IL-6: Interleukin-6
TGF-β1: transforming growth factor β1
Hpt: haptoglobin
PUFAs: Polyunsaturated fatty acids
GSH: Glutathione
ESCRT: Endosomal Sorting Complex Required for Transport
ACEIs: angiotensin-converting enzyme inhibitors
RFECV: Recursive Feature Elimination with Cross-Validation
AUC: Area Under the Curve
ROC: Receiver Operating Characteristic
AdaBoost: adaptive boosting
RF: random forest
SVM: support vector machine
CatBoost: Categorical gradient boosting machine
XGBoost: extreme gradient boosting classifier
SFS: Sequential Forward Selection
MI: mutual information
