# Supplementary material for: Machine learning-based prediction model for drug target identification and MASH improvement: a comprehensive analysis of biochemical and ferroptosis/autophagy biomarkers
Source: J Physiol Biochem. 2026 Apr 27;82(1):43. doi: 10.1007/s13105-026-01181-3 (PMC13121215; doi:10.1007/s13105-026-01181-3)

**Table S1.** showing stages of histological findings in NAFLD

| **Histologic findings** | **Stages** | | | | |
| --- | --- | --- | --- | --- | --- |
|  | **0** | **1** | **2** | **3** | **4** |
| **Steatosis** | <5% | 5-33% | 34-66% | >66% | - |
| **Intralobular inflammation** | None | <2 foci/ field (mild) | 2-4 foci/ field (moderate) | >4 foci/ field (sever) | - |
| **Hepatocyte ballooning** | Absent | Few cells | Many cells | - | - |
| **Mallory-Denk bodies** | Absent | Occasional | Several | - | - |
| **Fibrosis** | None | Perisinusoidal/ pericellular | + Periportal | + Focal bridging | Cirrhosis |

**Table S3.** Details of the GSE140994, GSE93819, and GSE8253 datasets were retrieved from the GEO database.

| **Accession number** | **Platform** | **Organism** | **Experiment type** | **Experimental design** | **Number of samples** | |
| --- | --- | --- | --- | --- | --- | --- |
|  | | | | | Case | Control |
| GSE140994 | GPL24557 [MoGene-2_0-st] Affymetrix Mouse Gene 2.0 ST Array [CDF:mogene20st_Mm_ENTREZG_22] | Mus musculus | Expression profiling by array | Liver sinusoidal endothelial cells (LSECs) form discontinuous, permeable microvessels with a distinct gene expression profile compared to continuous microvascular endothelial cells, such as those in the lung. LSECs play a crucial role in liver fibrogenesis in the CDAA dietary model of non-alcoholic steatohepatitis (NASH). Microarrays were used to analyze gene expression in murine liver endothelial cells after 10 weeks on a CDAA diet. Murine liver sinusoidal endothelial cells (mLSECs) from Gata4 endothelial cell-subtype specific knockout (Gata4-LSEC-KO) and sibling control mice were isolated for RNA extraction and hybridization on Affymetrix microarrays | 5 | 5 |
| GSE93819 | GPL1261 [Mouse430_2] Affymetrix Mouse Genome 430 2.0 Array | Mus musculus | Expression profiling by array | 7-week-old C57BL/6J male mice were fed normal chow (CRF-1) or CL diet (60% calories from fat, 1.25% cholesterol, and 0.5% sodium cholate) for 12 weeks. | 5 | 5 |
| GSE8253 | [GPL85](https://www.ncbi.nlm.nih.gov/geo/query/acc.cgi?acc=GPL85)[RG_U34A] Affymetrix Rat Genome U34 Array | Rattus norvegicus | Expression profiling by array | Two groups of male Sprague dawley rats were fed liquid diets via total enteral nutrition.  Group 1, Control, Rats were fed diets containing 5% Corn oil at 187 Kcal/kg3/4/d for 3 weeks.  Group 2, NASH, Rats were fed diets containing 70% corn oil at 220 Kcal/kg3/4/d for 3 weeks. | 3 | 3 |

**Table S4.** List of primer assays.

| Gene | Gene globe ID |
| --- | --- |
| Rn_GAPDH_1_SG (NM_002046) | QT00079247 |
| Rn_GPX4_1_SG (NM_001039849 (1037 bp), NM_017165 (923 bp))Amplicon Length  104 (NM_001039849),104 (NM_017165) | **QT00174853** |
| Rn_LPCAT3_1_SG (NM_005768).  NM_001012189, XM_342754 | QT01569176 |
| Hs_ACSL4_1_SG (NM_053623, XM_008773385, XM_006257315, XM_006257316, XM_006257314).) | QT00190708 |
| Rn_TSG101_1_SG ((NM_181628)) | QT0017752 |
| Rn_HGS_1_SG (NM_019387, XM_006247909, XM_006247908). | QT00366779 |
| Rn_SNF8_1_SG (NM_001007804, XM_006247185). | QT00453488 |
| Rn_ACTB_1_SG ((NM_031144)). | QT00193473 |
| rno-miR-329-5p (MIMAT0017031) | ZP00004395 |
| rno-miR-23a-5p(MIMAT0004496) | YP00205631 |
| rno-miR-27a-5p(MIMAT0004501) | YP00206021 |
| SNORD44 | YP00203902 |
| LINC00442 (ENST00000428086) | LPH09990A |
| CTBP1-AS2 (NR_033339) | LPH01850A |

**Table S5.** Shows the number of samples per normal, disease model, and treatment groups.

| **Condition** | **Number of samples** |
| --- | --- |
| Normal (Healthy) | 35 |
| MASH-12 weeks | 35 |
| Febuxostat-1.5 | 10 |
| Febuxostat-3 | 10 |
| Febuxostat-6 | 10 |
| Amlodipine | 10 |
| Perindopril | 10 |
| Amlodipine/Perindopril | 10 |
| atorvastAtin-20 | 10 |

**Table S6.** Molecular, Biochemical, and immunohistochemical features used in ML models.

| **Molecular (11 features)** | **Biochemical (12 features)** | **Immunohistochemistry (6 features)** |
| --- | --- | --- |
| 1. GPX4 mRNA 2. LPCAT3 mRNA 3. ACSL4 mRNA 4. HGS mRNA 5. TSG101 mRNA 6. SNF8 mRNA 7. rno-miR-329-5p 8. rno-miR-23a-5p 9. rno-miR-27a 10. LINC00442 11. CTBP1-AS2 | 1. ALT 2. AST 3. ALP 4. GGT 5. Total Bilirubin 6. Direct Bilirubin 7. AFP 8. Albumin 9. TC 10. TG 11. LDL-C 12. HDL-C | 1. Hpt (ug/gm) 2. TMAO (ng/mg)  3. GPX4 (ng/mg) 4. TSG101 (ng/mg) 5. IL-6 (ρg/g)  6. TGFβ1 (ρg/g) |

**Table S7.** Pathology assessment of liver biopsies from rats among control, MASH, and treatment groups.

|  | **Control**  **(n=35)** | **MASH(n=35)** | **Febuxostat-1.5 (n=10)** | **Febuxostat-3(n=10)** | **Febuxostat-6(n=10)** | **Amlodipine(n=10)** | **Perindopril(n=10)** | **Amlodipine/Perindopril(n=10)** | **Atorvastatin-20(n=10)** | **P-value** |
| --- | --- | --- | --- | --- | --- | --- | --- | --- | --- | --- |
| **NAS score** | | | | | | | | | | 0.000 |
| **0** | 100% |  |  |  | 30% | **-** | **-** | 30% | **-** |  |
| **1** | - | - | - | - | 40% | **-** | **-** | 30% | **-** |  |
| **2** | - | - | - | 20% | 30% | **-** | 60% | 40% | **-** |  |
| **3** | - | - | - | 50% | **-** | **-** | 40% | **-** | **-** |  |
| **4** | - | - | 60% | 30% | **-** | 50% | - | **-** | **-** |  |
| **5** | - | - | 40% | - | **-** | 50% | **-** | **-** | 70% |  |
| **6** | - | 60% | - | - | **-** |  | **-** | **-** | 30% |  |
| **7** | - | 40% | - | - | **-** | **-** | **-** | **-** | **-** |  |
| **Steatosis** | | | | | | | | | | 0.000 |
| **0:<5%** | 100% | - | 20% | 50% | 80% | 10% | 30% | 60% | 10% |  |
| **1: 5-33%** | - | 8.57% | 50% | 40% | 10% | 20% | 40% | 20% | 10% |  |
| **2: 34-66%** | - | 34.29% | 30% | 10% | 10% | 30% | 20% | 10% | 50% |  |
| **3: >66%** | - | 57.14% | - | - | - | 40% | 10% | 10% | 30% |  |
| **Intralobular Inflammation** | | | | | | | | | | 0.000 |
| **0: none** | 100% | - | 30% | 40% | 50% | 20% | 20% | 70% | 20% |  |
| **1:<2 mild** | - | 5.7% | 50% | 50% | 40% | 50% | 70% | 20% | 60% |  |
| **2:2-4 moderate** | - | 25.8% | 20% | 10% | 10% | 20% | 10% | 10% | 20% |  |
| **3:>4 severe** | - | 68.5% | - | - | - | 10% | - | - | - |  |
| **Ballooning** | | | | | | | | | | 0.000 |
| **0: absent/none** | 100% | - | 10% | 40% | 50% | 10% | 20% | 40% | 10% |  |
| **1: few** | - | 11.43% | 40% | 30% | 50% | 50% | 40% | 40% | 20% |  |
| **2: many** | - | 88.57% | 50% | 30% | - | 40% | 40% | 20% | 70% |  |
| **Mallory-Denk bodies** | | | | | | | | | | 0.000 |
| **0:** **Absent** | 100% | - | 60% | 50% | 70% | 10% | 30% | 50% | 10% |  |
| **1: rare or occasional** | - |  | 40% | 50% | 30% | 50% | 50% | 40% | 20% |  |
| **2:** **Several** | - | 100% | - | - | - | 40% | 20% | 10% | 70% |  |
| **Fibrosis** | | | | | | | | | | 0.000 |
| **0: Absent/ none** | 94.29% | - | 30% | 50% | 80% | 10% | 20% | 60% | 10% |  |
| **F1:** **perisinusoidal/ pericellular** | 5.71% | 14.28% | 40% | 40% | 10% | 30% | 20% | 20% | 20% |  |
| **F2: + periportal** | - | 51.43% | 30% | 10% | 10% | 50% | 50% | 20% | 30% |  |
| **F3: +focal Bridging** | - | 34.29% | - | - | - | 10% | 10% | - | 40% |  |
| **F4:** **Cirrhosis** | - | - | **-** | - | **-** | - | - | - | - |  |

**Table S8.** Show the included and excluded features for each feature group**.**

| **Model** | **Included Features** | **Excluded Features** |
| --- | --- | --- |
| **Molecular**  Included: 9  Excluded: 2  Total: 11 | LPCAT3 mRNA ACSL4 mRNA HGS mRNA TSG101 mRNA SNF8 mRNA rno-miR-27a-5p  rno-miR-329-5p  LINC00442 CTBP1-AS2 | GPX4 mRNA  rno-miR-23a-5p |
| **Biochemical**  Included: 11  Excluded: 1  Total: 12 | ALT AST ALP GGT T.Bilirubin D.Bilirubin  AFP  Albumin  TC  TG  LDL-C | HDL-C |
| **Immunohistochemical**  Included: 5  Excluded: 1  Total: 6 | Hpt (ug/gm) TMAO (ng/mg) GPX4 (ng/mg) TSG101 (ng/mg) TGFβ1 (ρg/g) | IL-6 (ρg/g) |
| **Combined**  Included: 16  Excluded: 13  Total: 29 | LPCAT3 mRNA HGS mRNA TSG101 mRNA SNF8 mRNA rno-miR-27a-5p rno-miR-329-5p CTBP1-AS2 TMAO (ng/mg) GPX4 (ng/mg) TGFβ1 (ρg/g) ALT  AST ALP GGT D.Bilirubin Albumin | GPX4 mRNA  ACSL4 mRNA  rno-miR-23a-5p  LINC00442  T.Bilirubin  AFP  TC  TG  LDL-C  HDL-C  Hpt (ug/gm)  IL-6 (ρg/g)  TSG101 (ng/mg) |

**Figure S1.** Gene Ontology of the retrieved genes by GeneCards (<https://www.genecards.org/>, accessed May 2022).


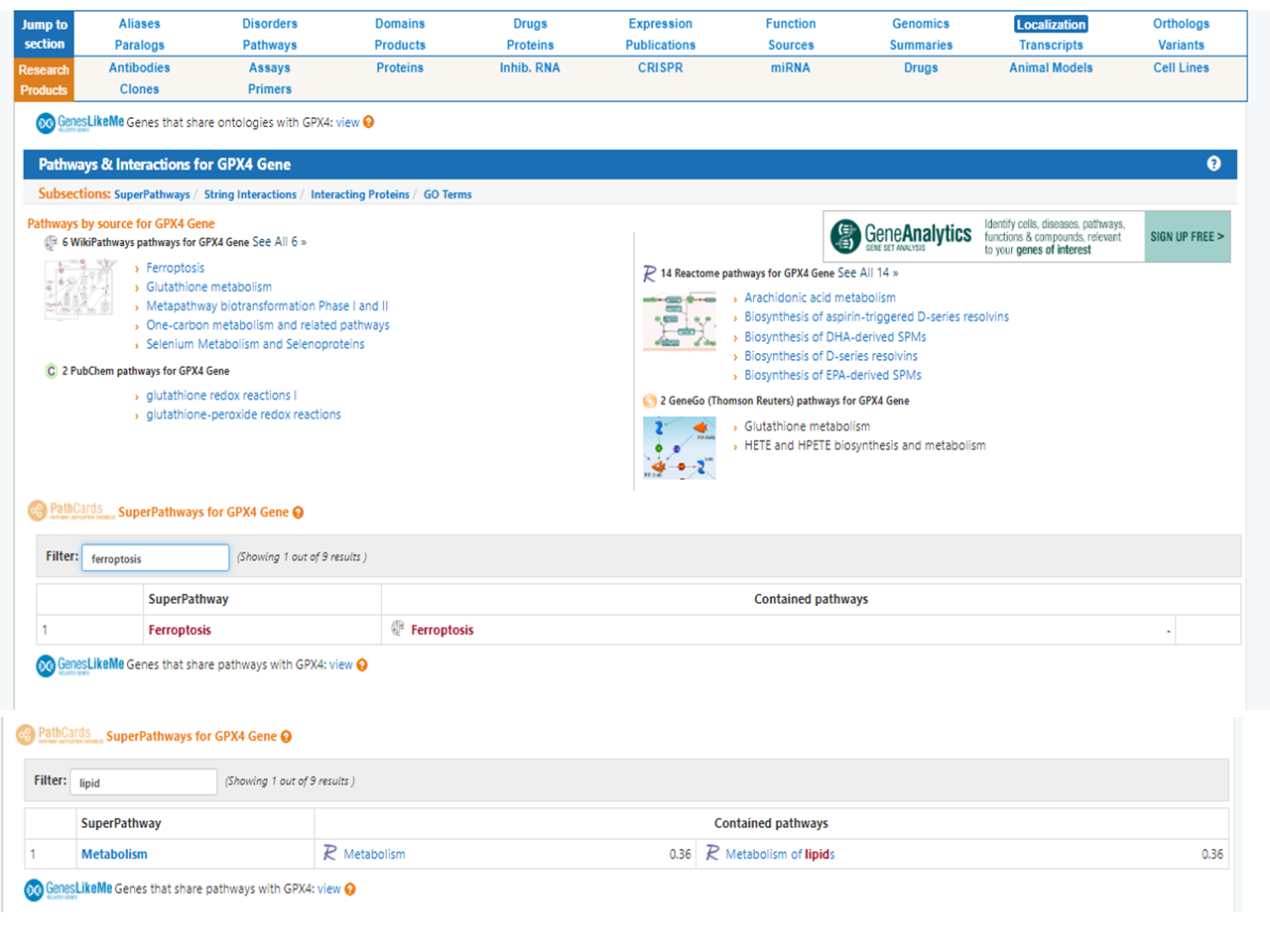


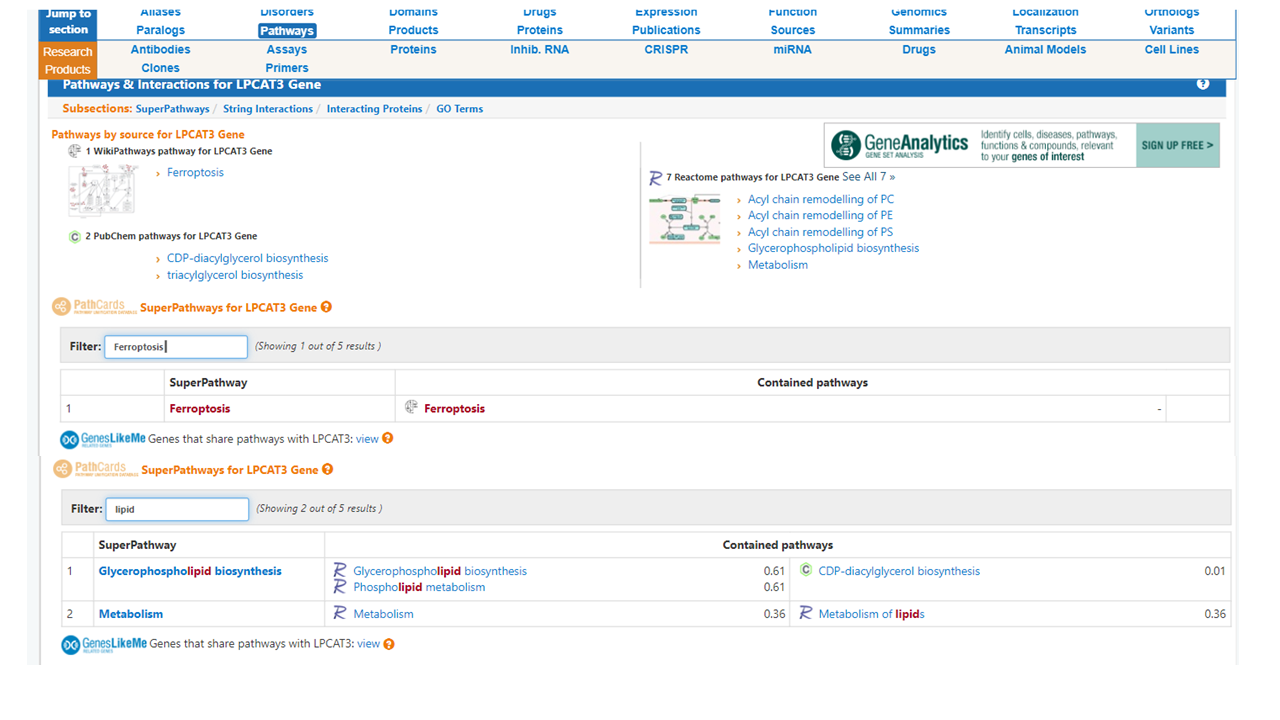


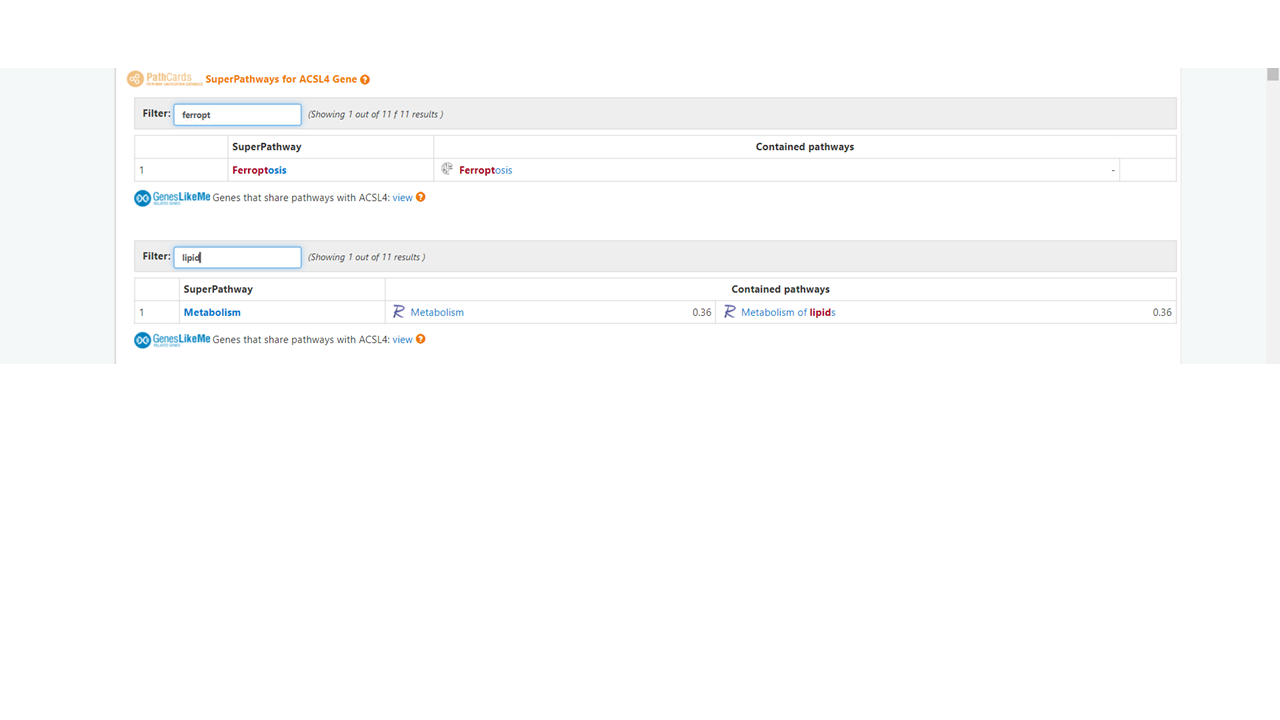


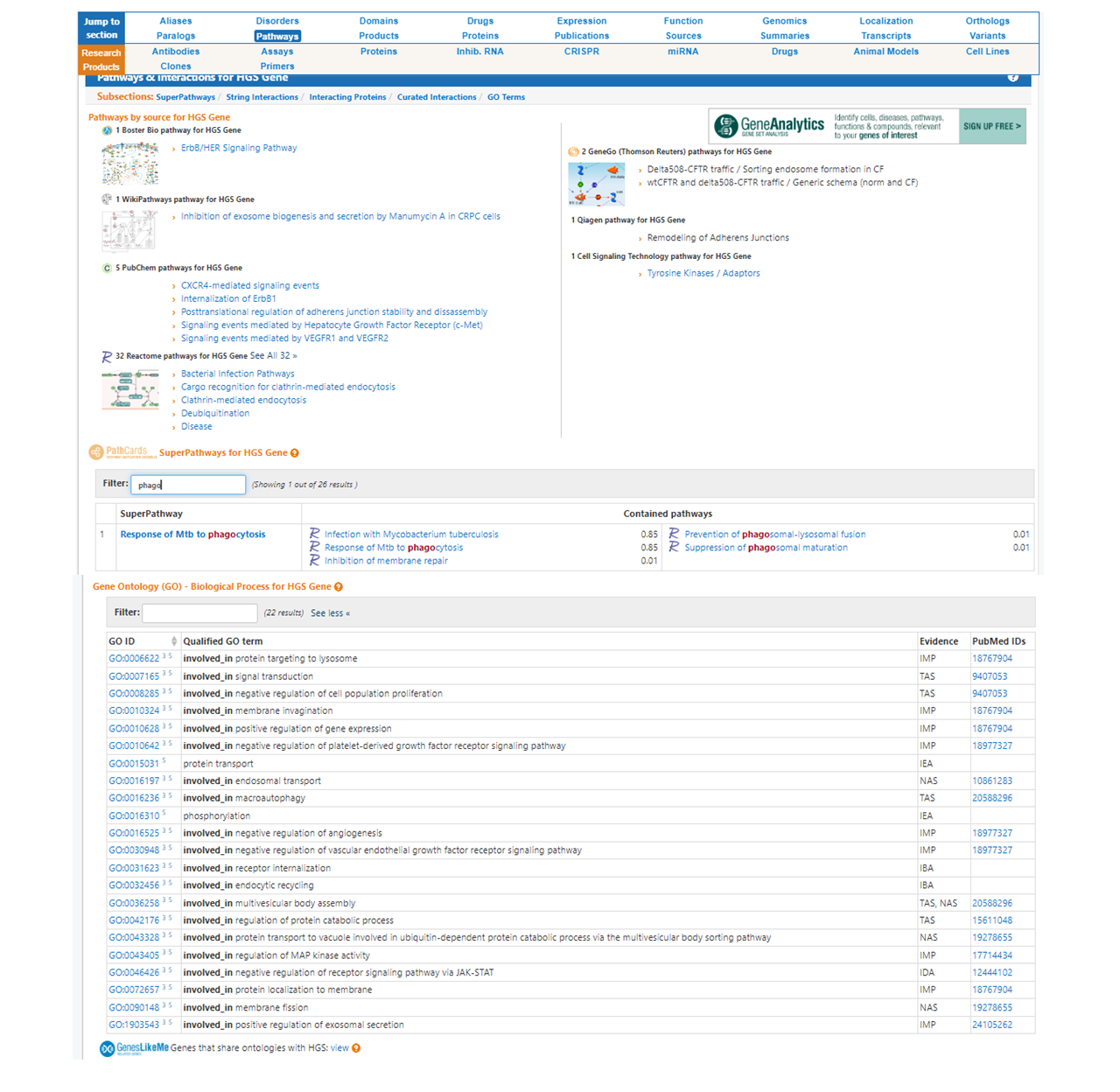


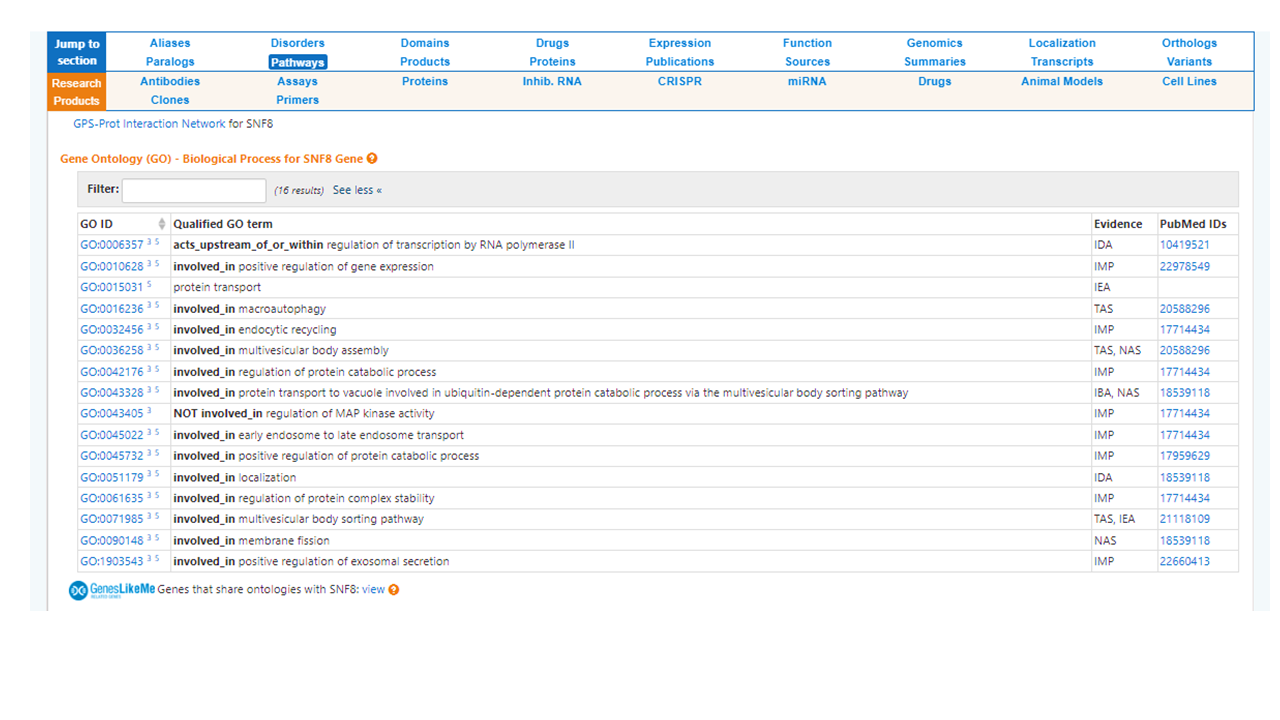


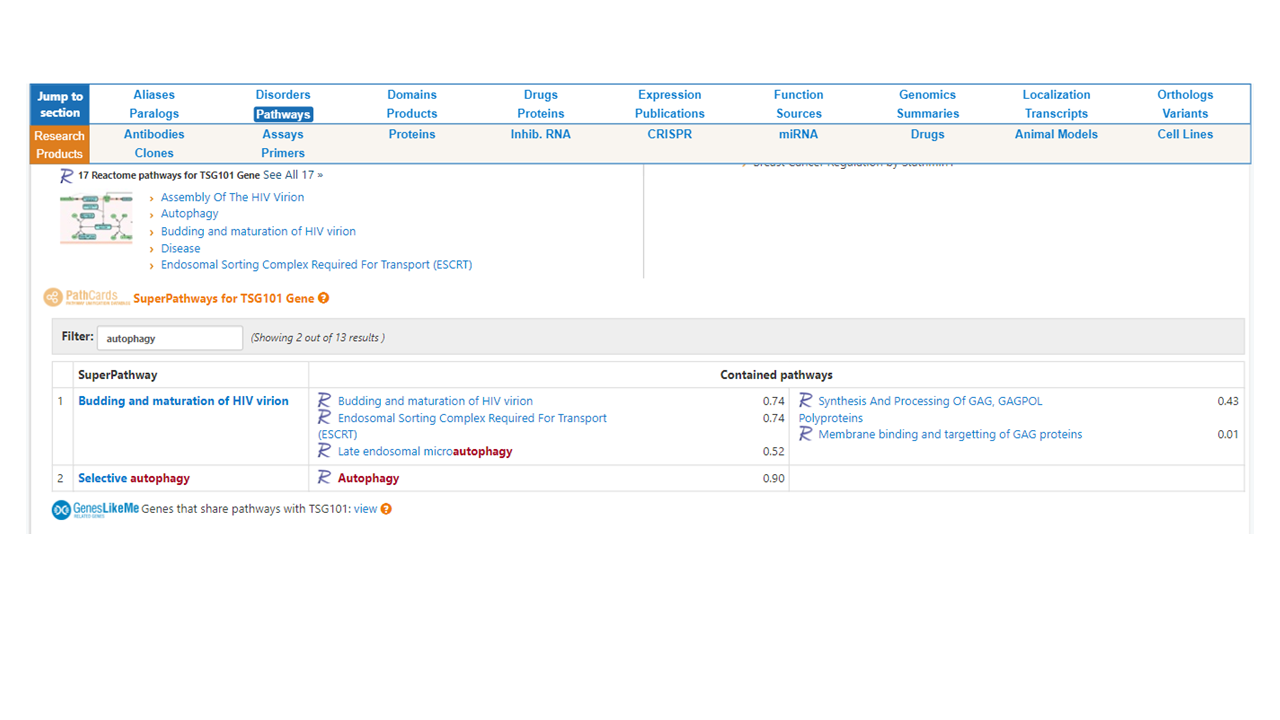


**Figure S2.** Protein Interaction analysis of the retrieved genes.


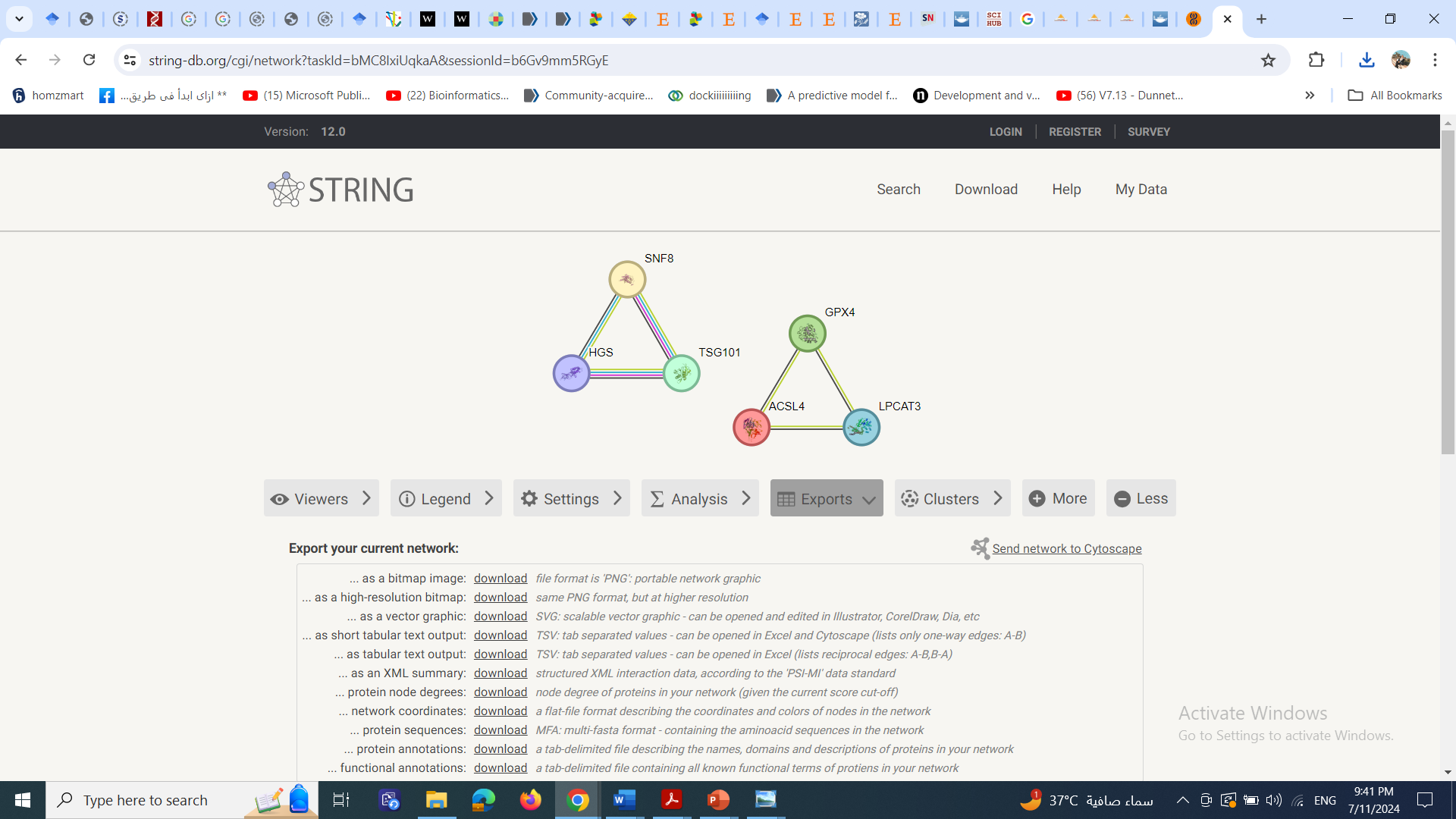


**Figure S3.**Validation of the interaction between the selected mRNAs and the retrieved miRNAs from the mirWalk (<http://mirwalk.umm.uni-heidelberg.de/>)


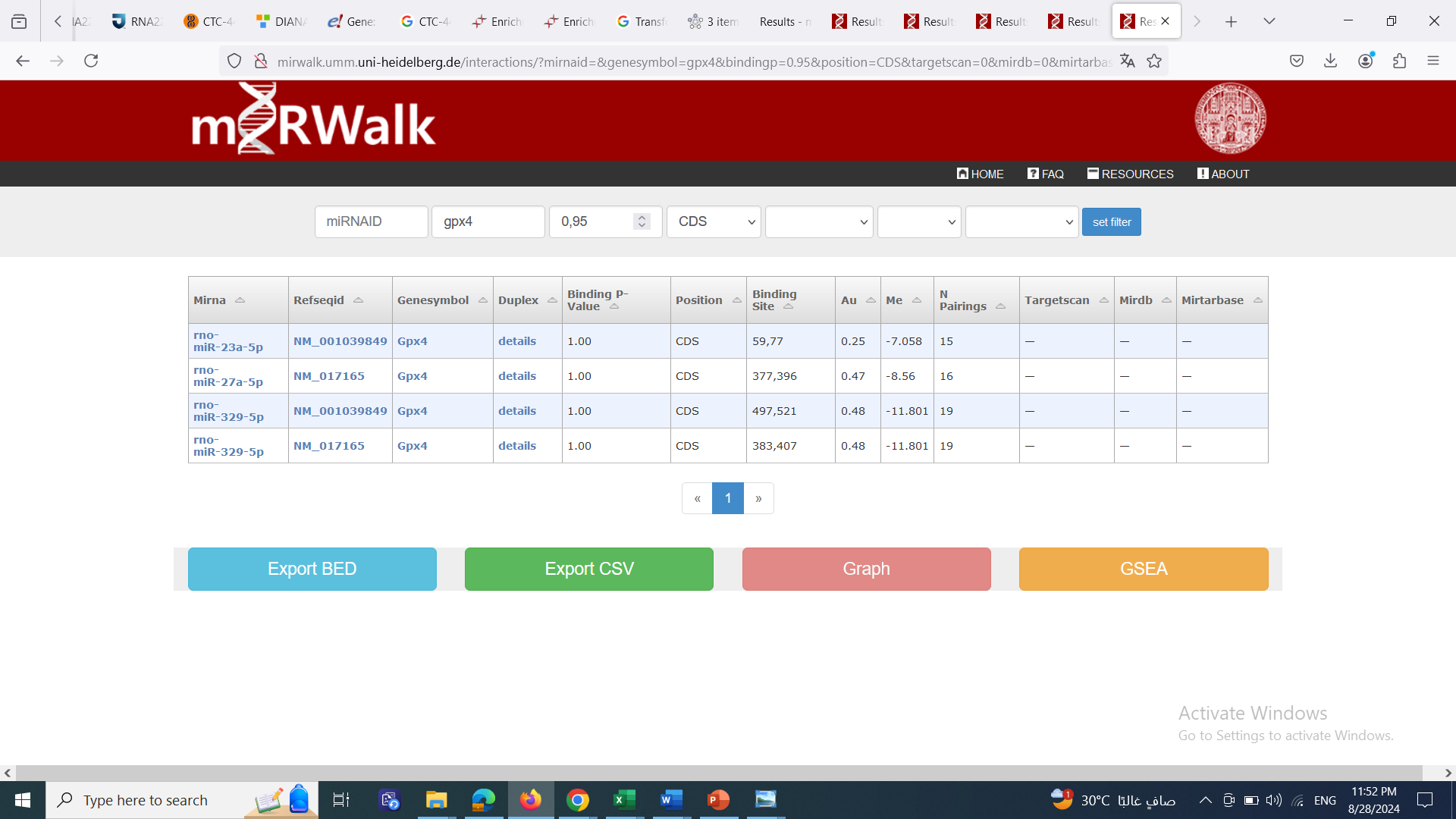


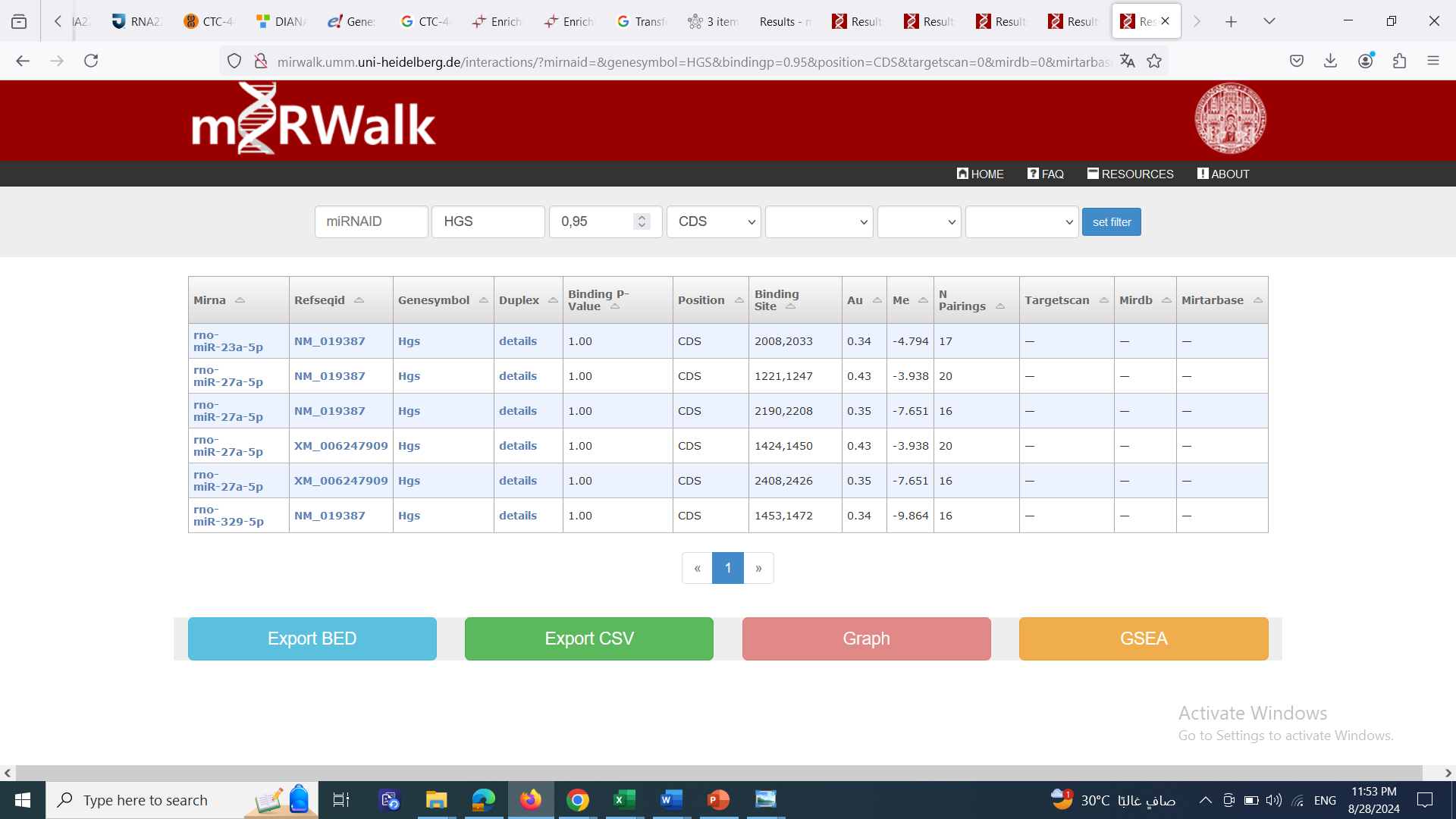


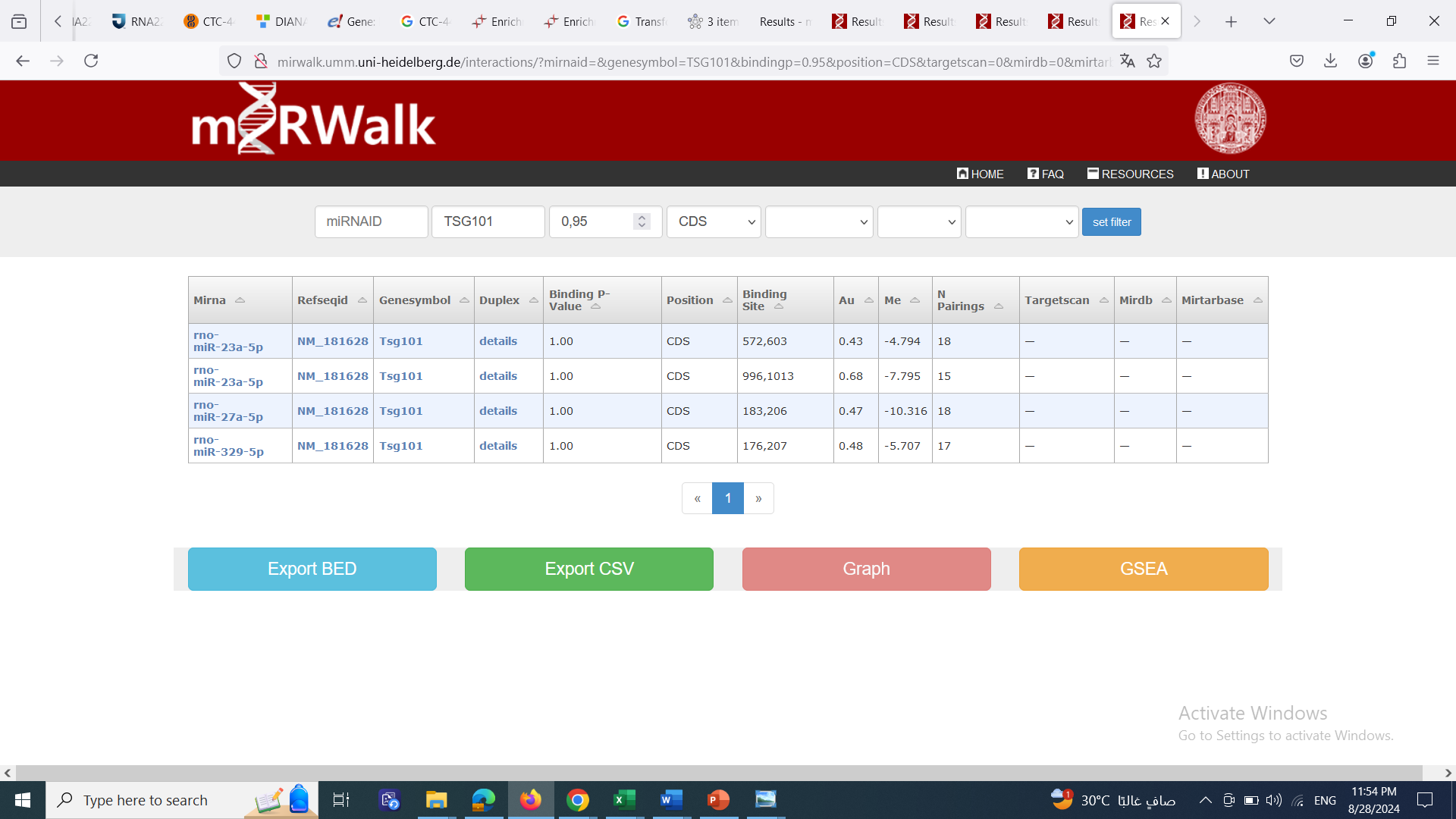


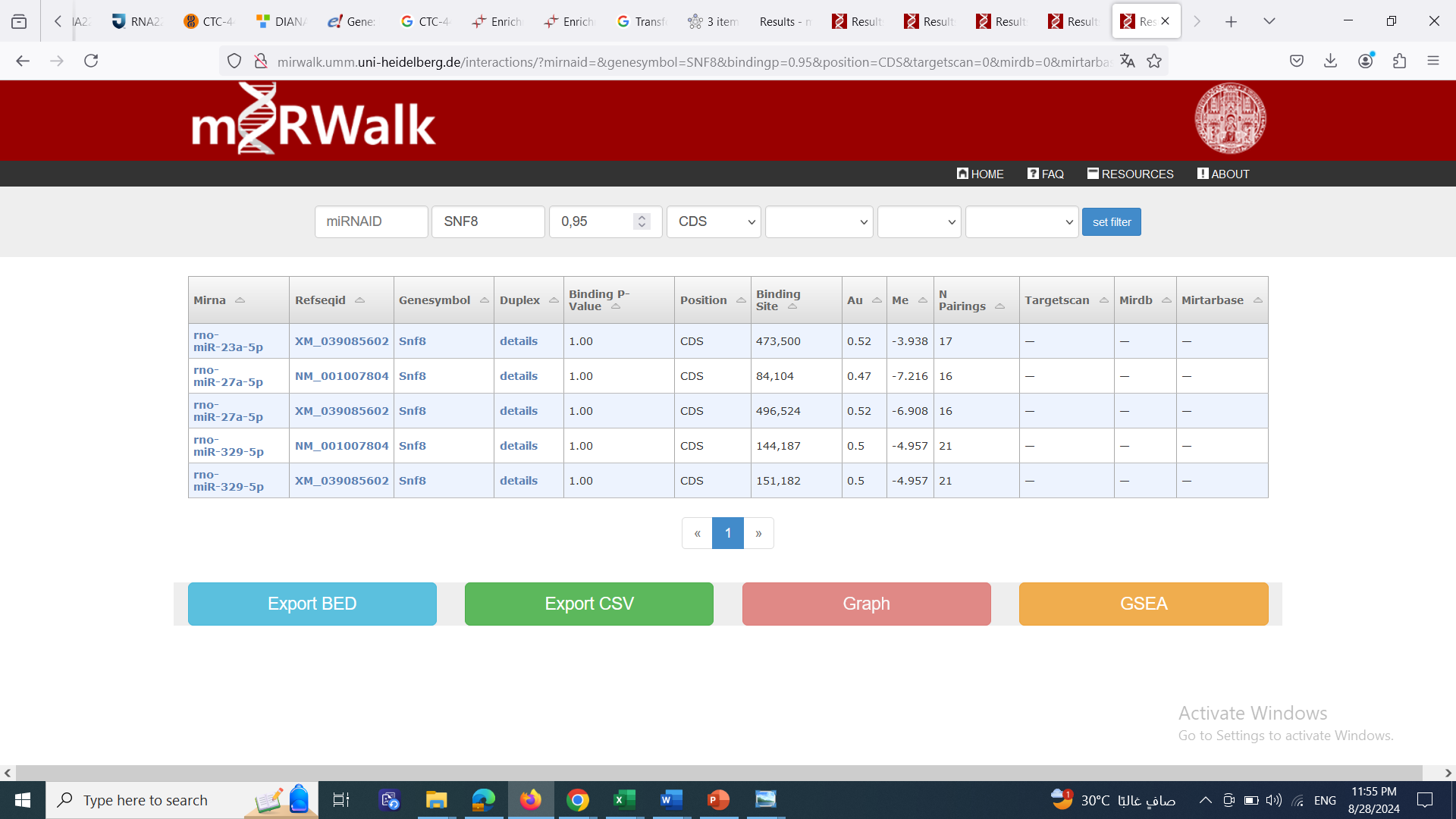


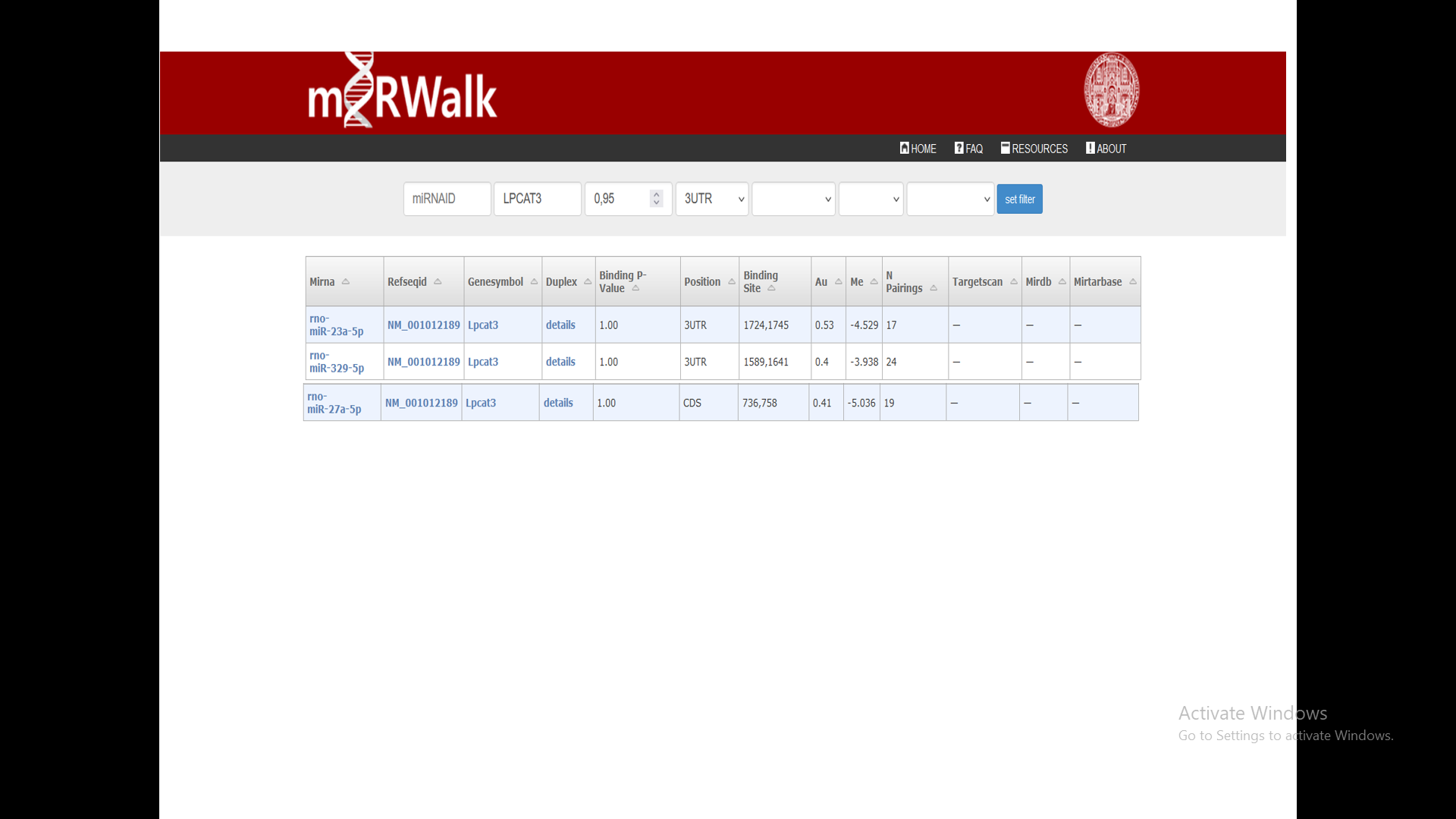


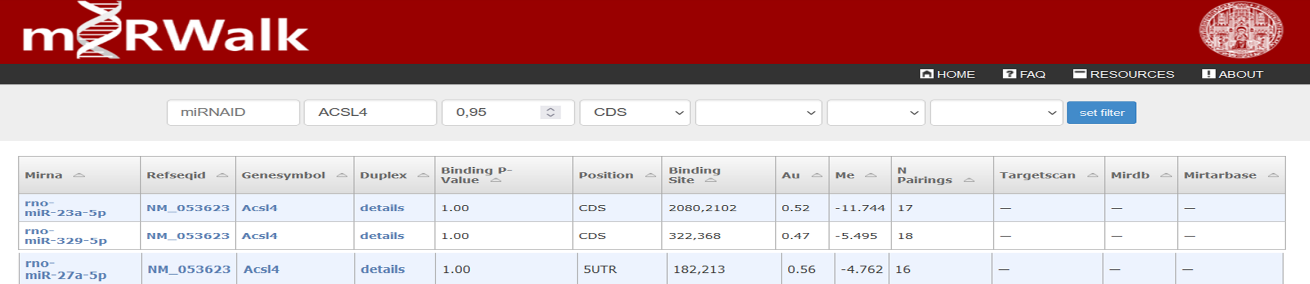


**Figure S4:** Validation of the interaction between the retrieved miRNAs and LncRNAs by RNA22 (<https://cm.jefferson.edu/rna22/>, accessed May 2022) database.

rno-miR-23a-5p+ LINC00442


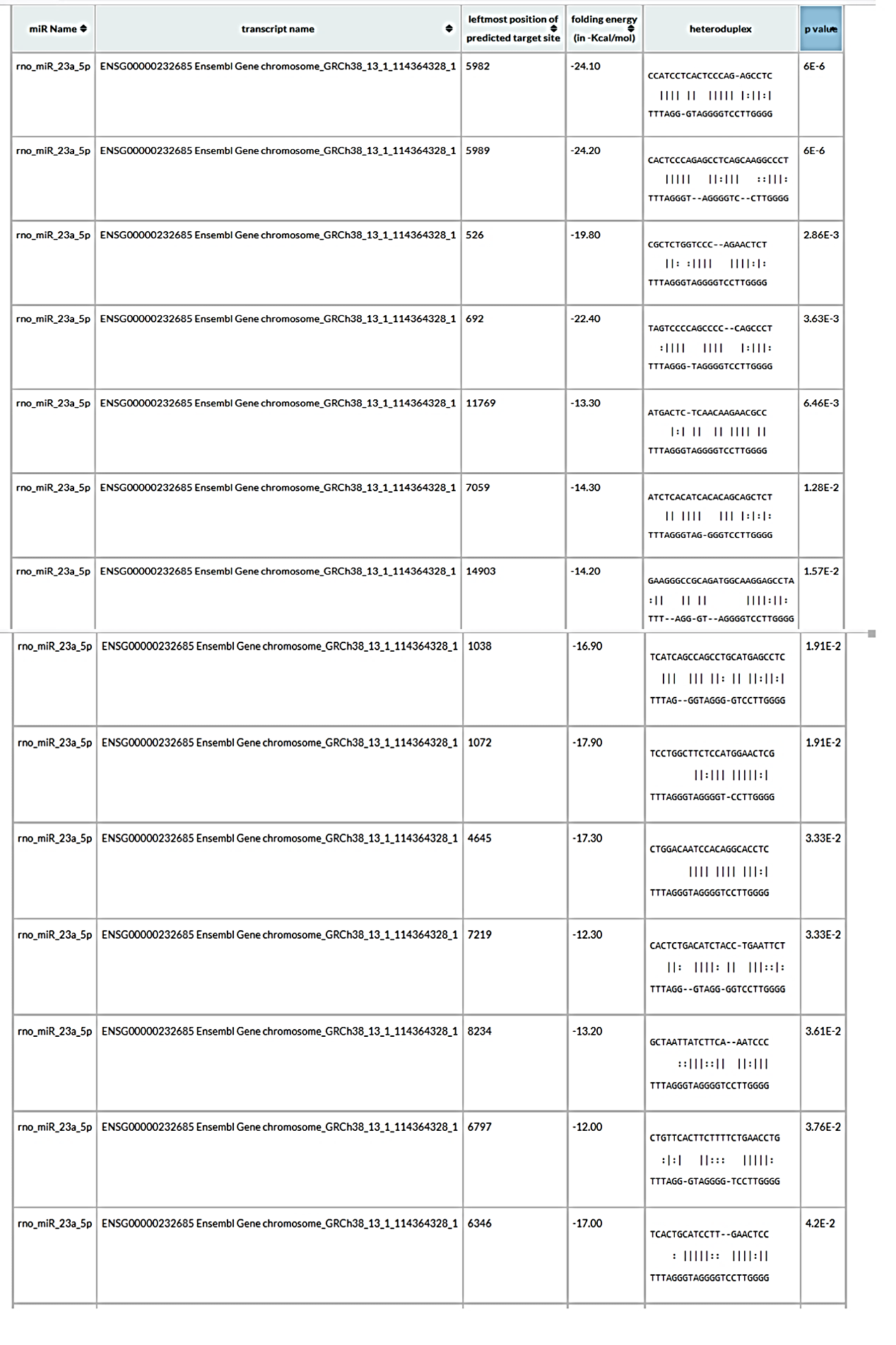


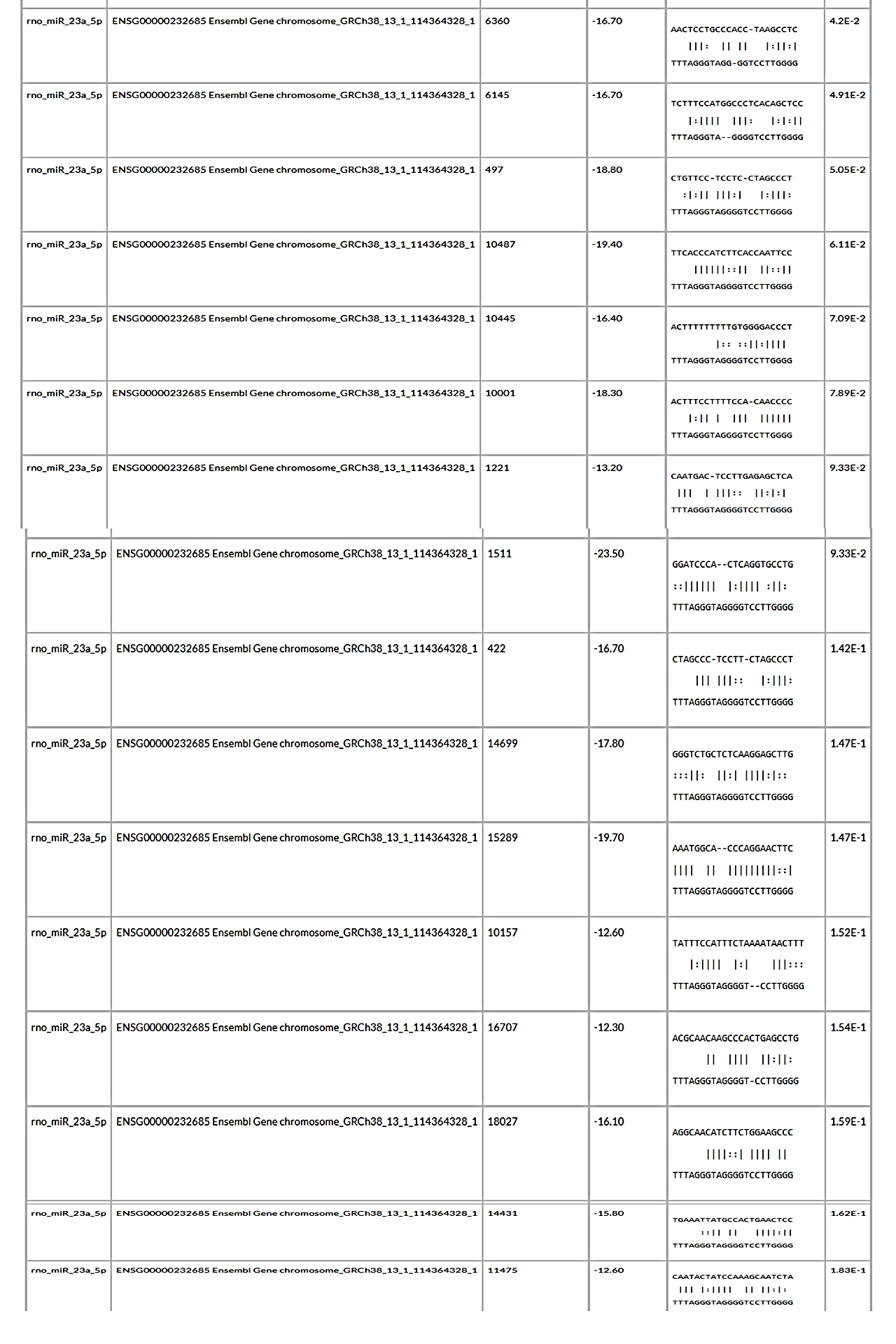


rno-miR-23a-5p+ CTBP1-AS2


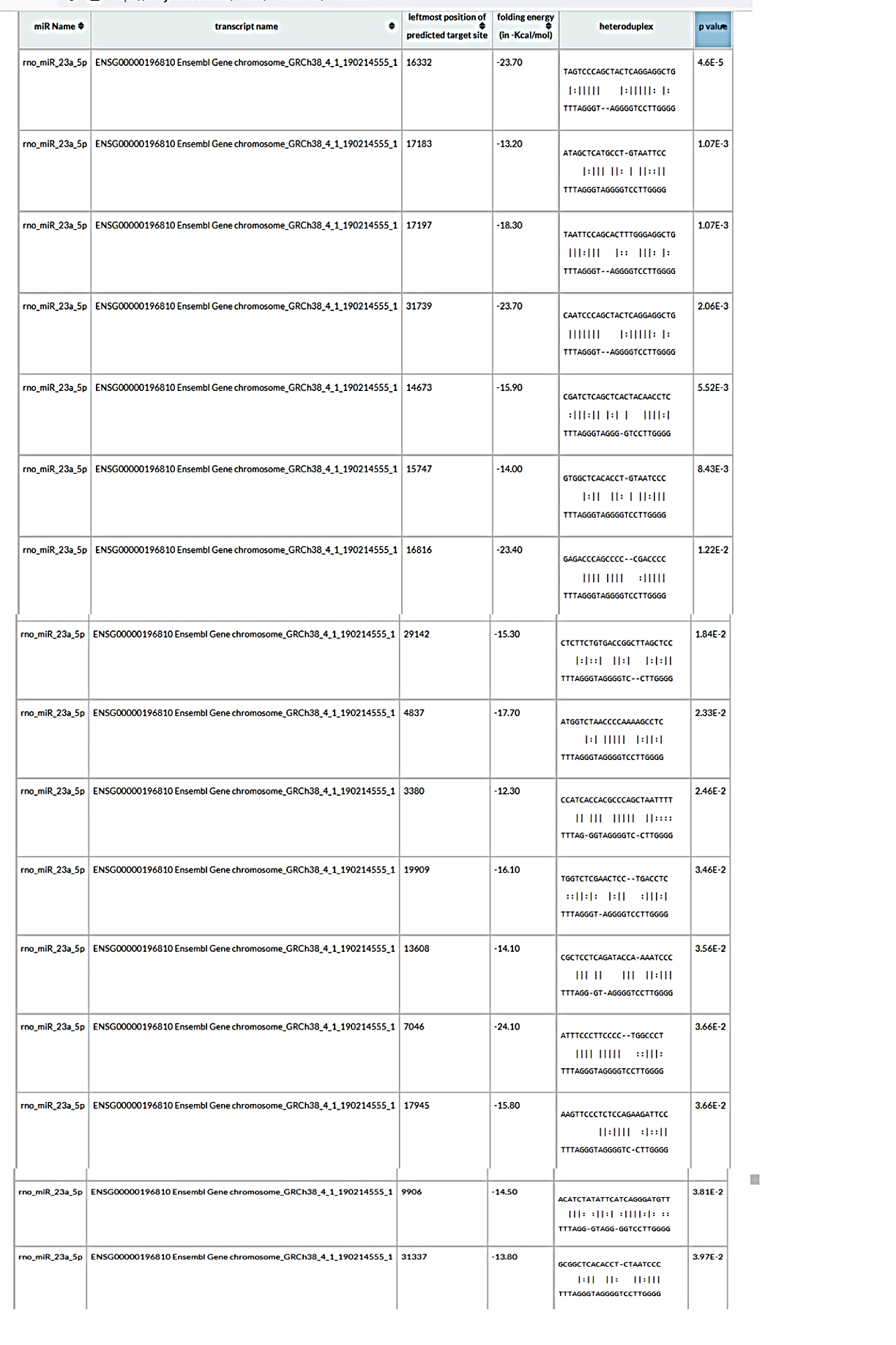


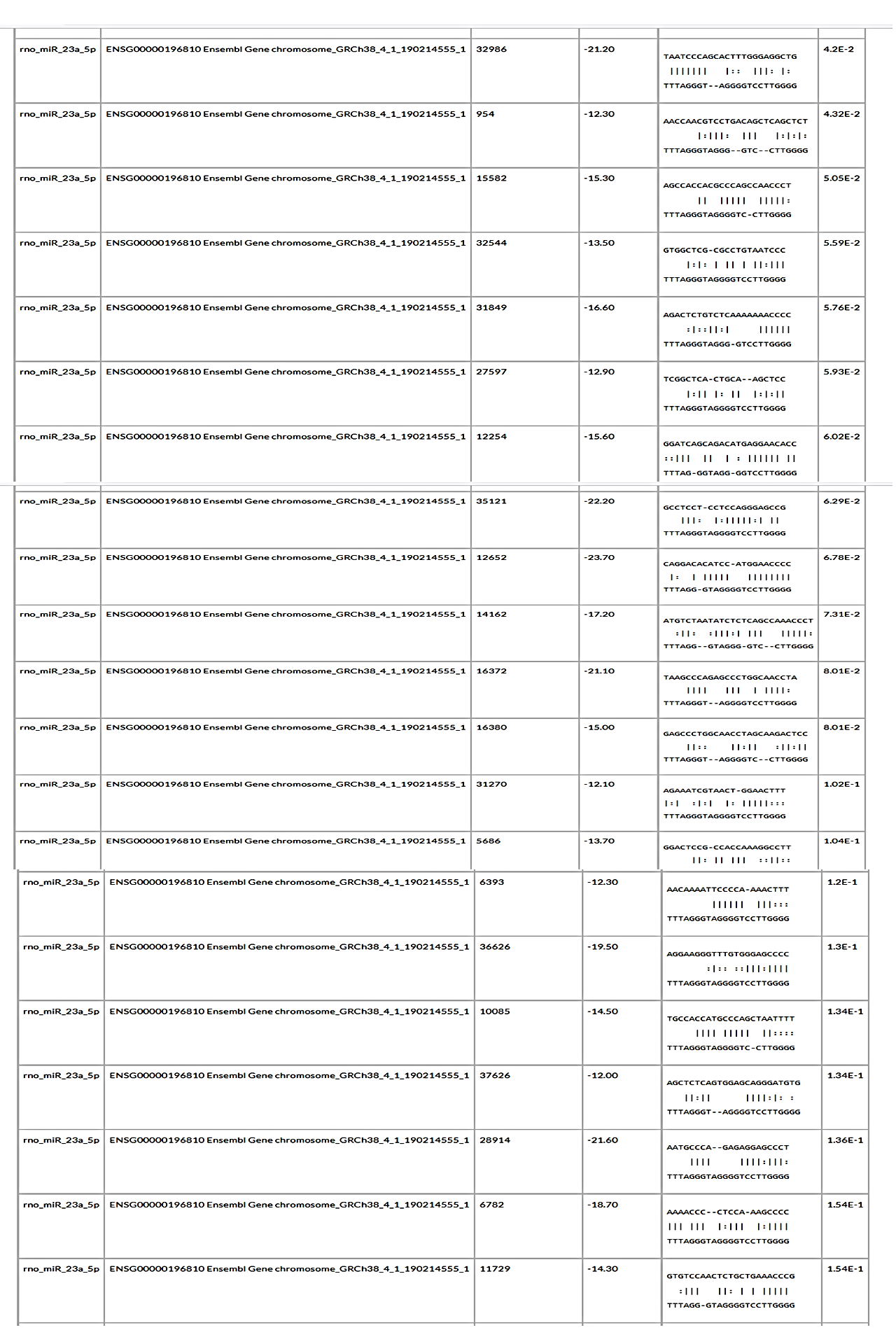


rno-mir-27a+ LINC00442


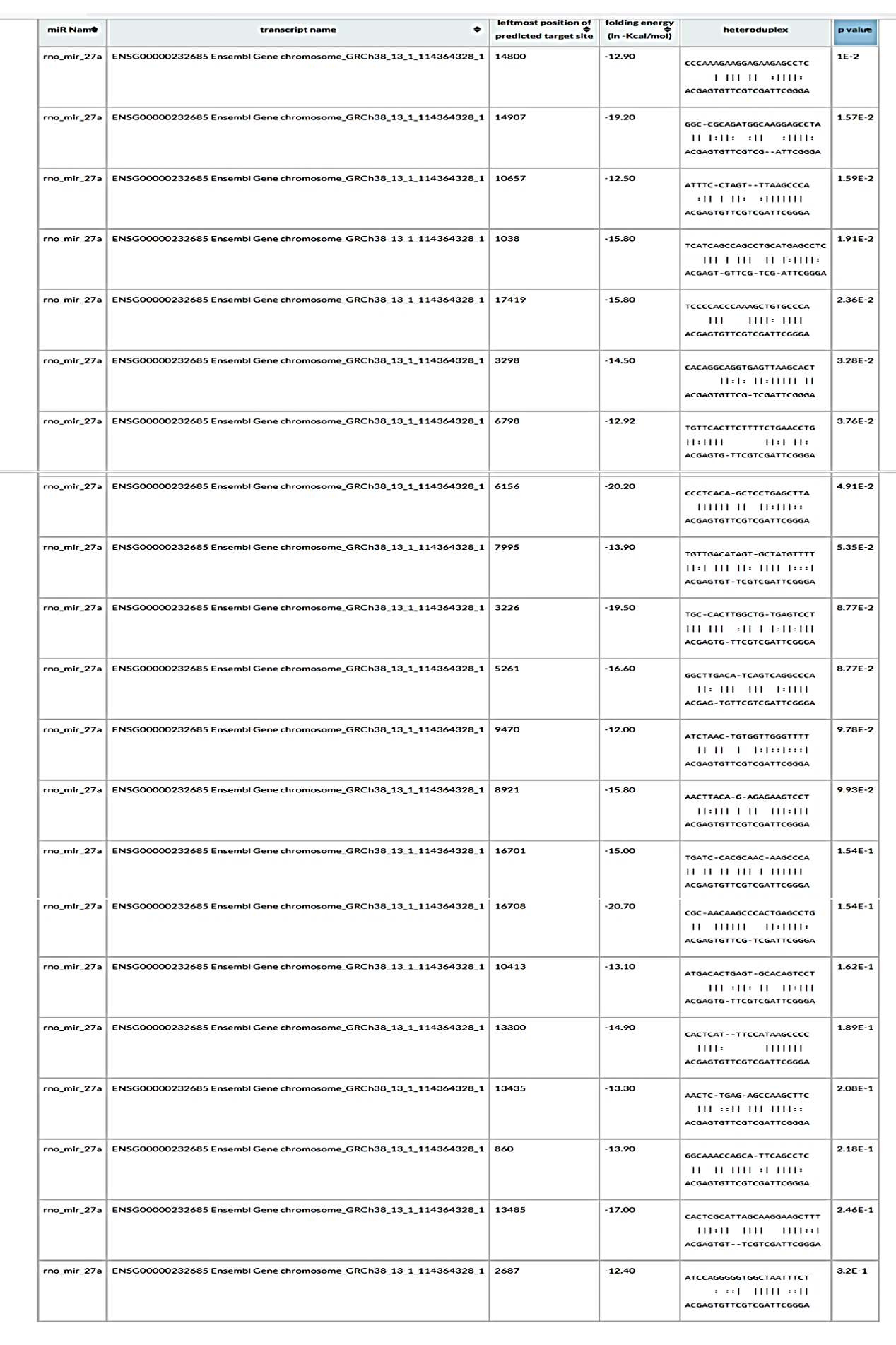


rno-mir-27a+ CTBP1-AS2


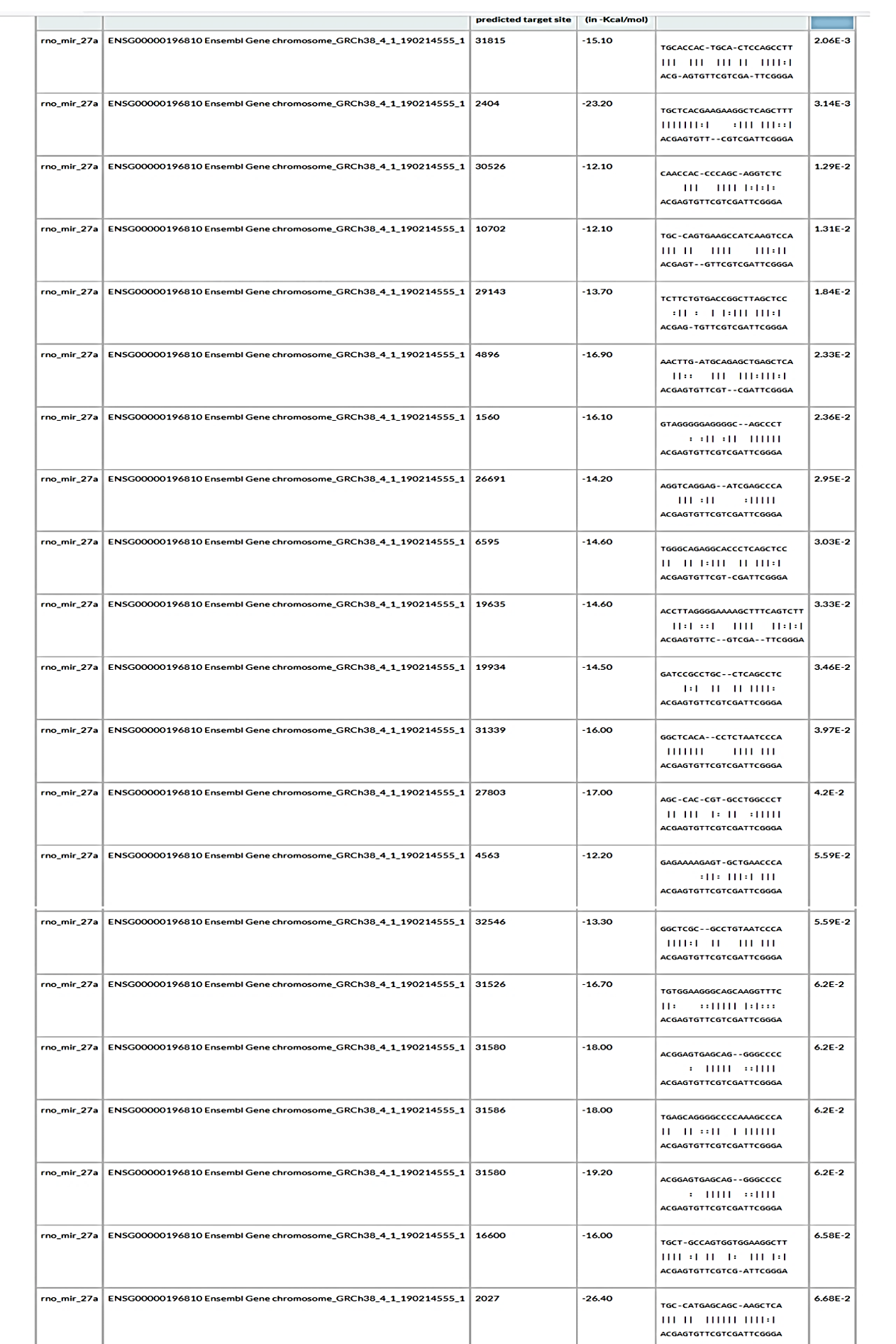


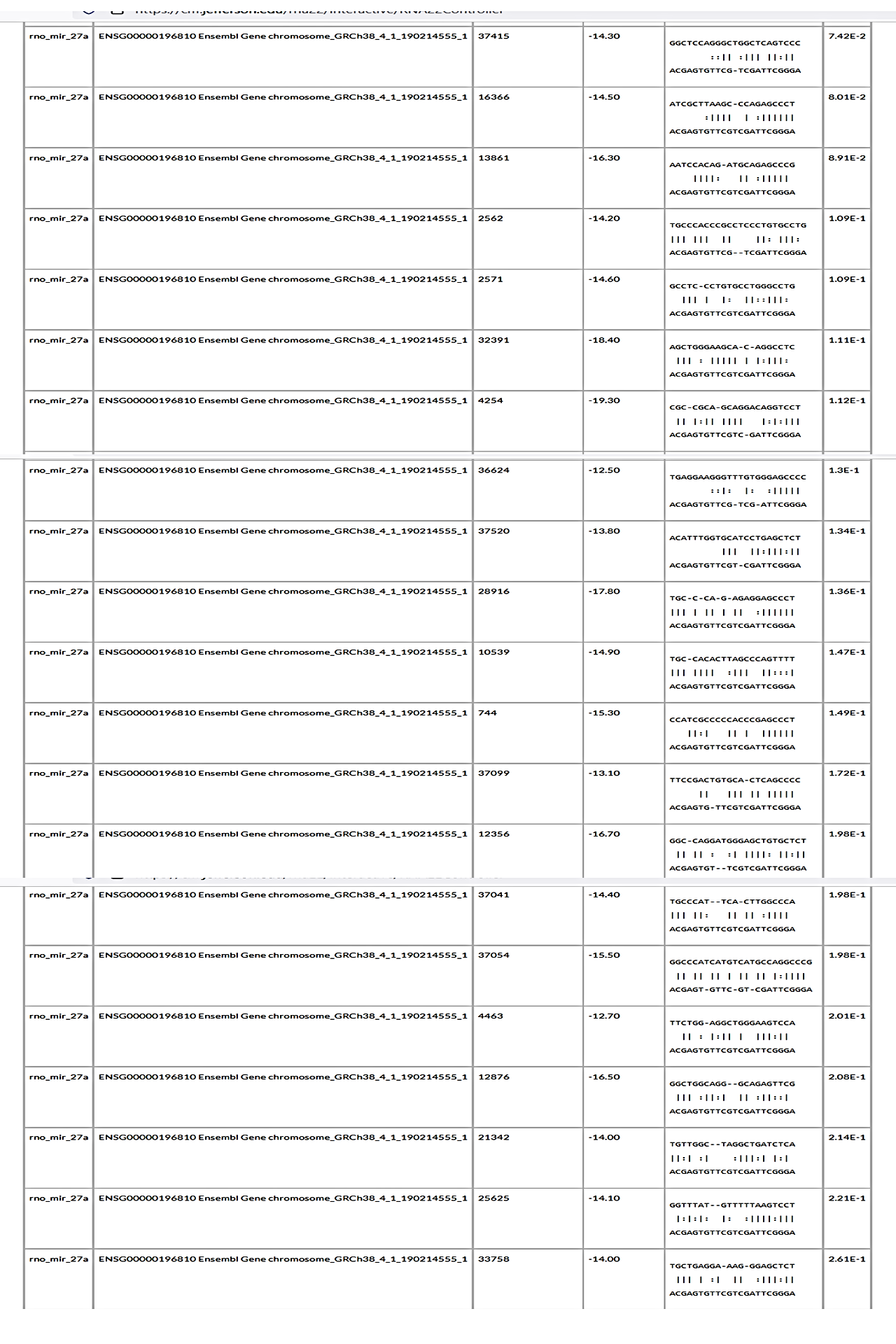


rno-miR-329-5p+ LINC00442


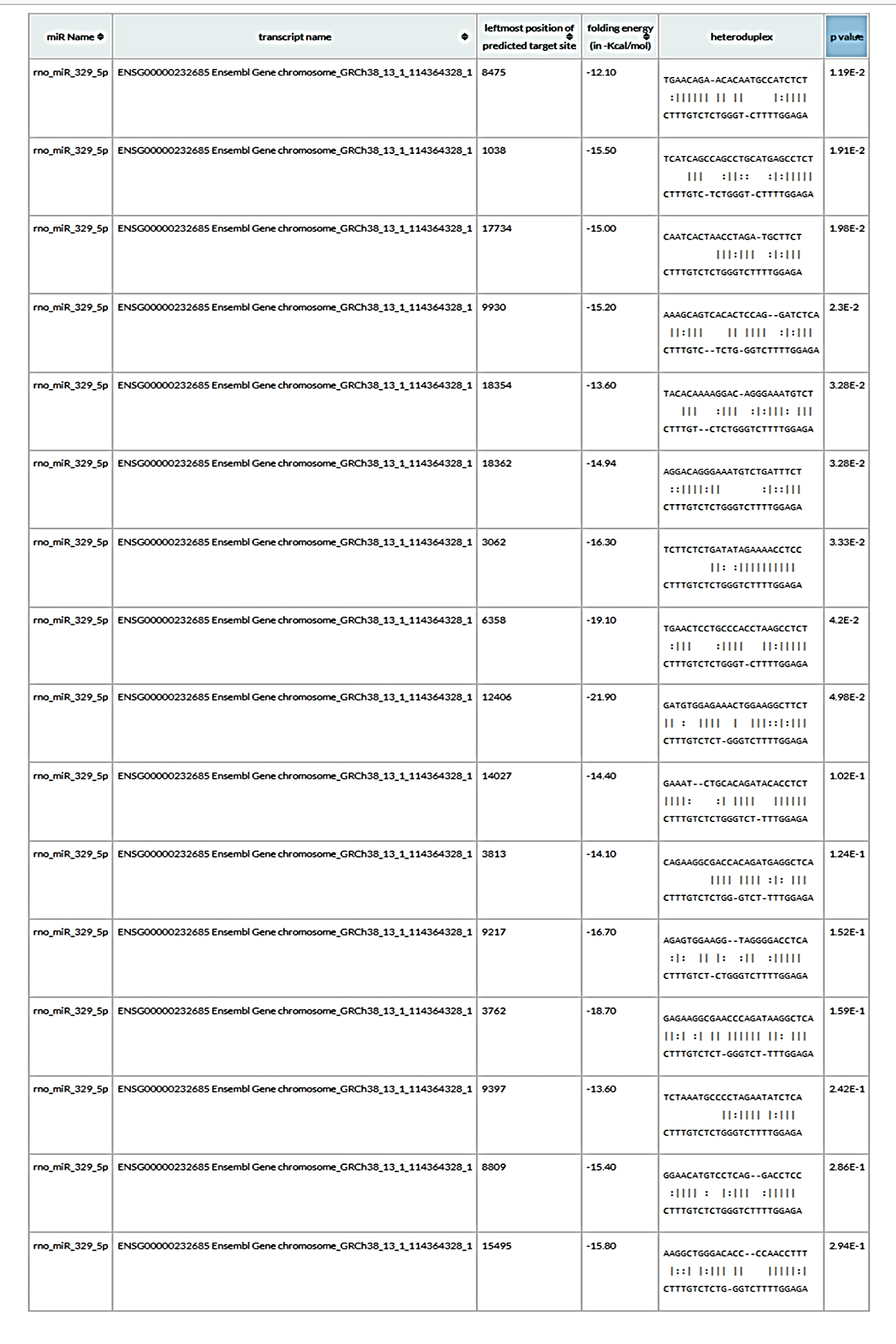


rno-miR-329-5p+ CTBP1-AS2


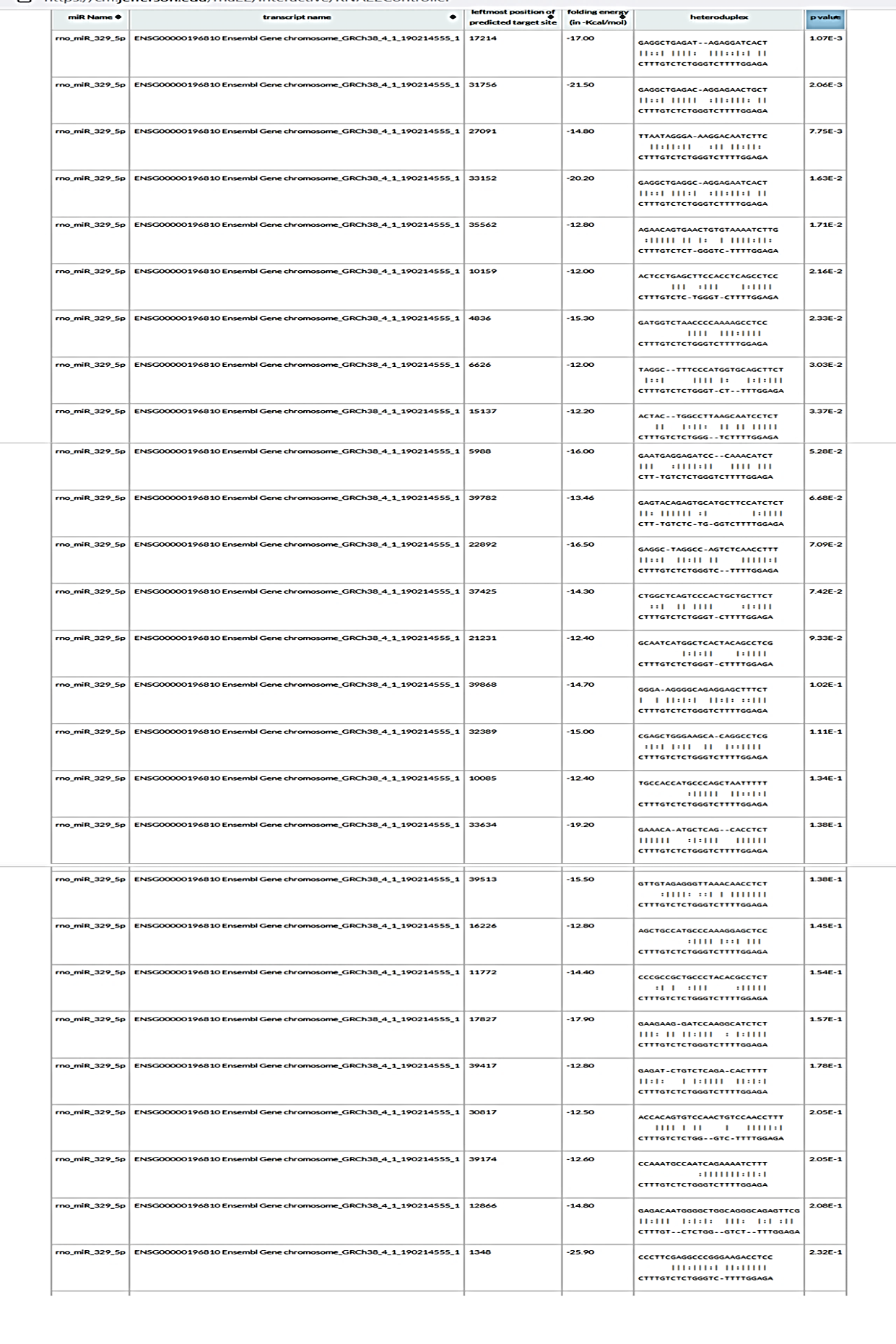


**Figure S5**. Displays the number of improved cases in each class: Massive Improved (58), Medium Improved (33), Light Improved (28), and Not Improved (21).


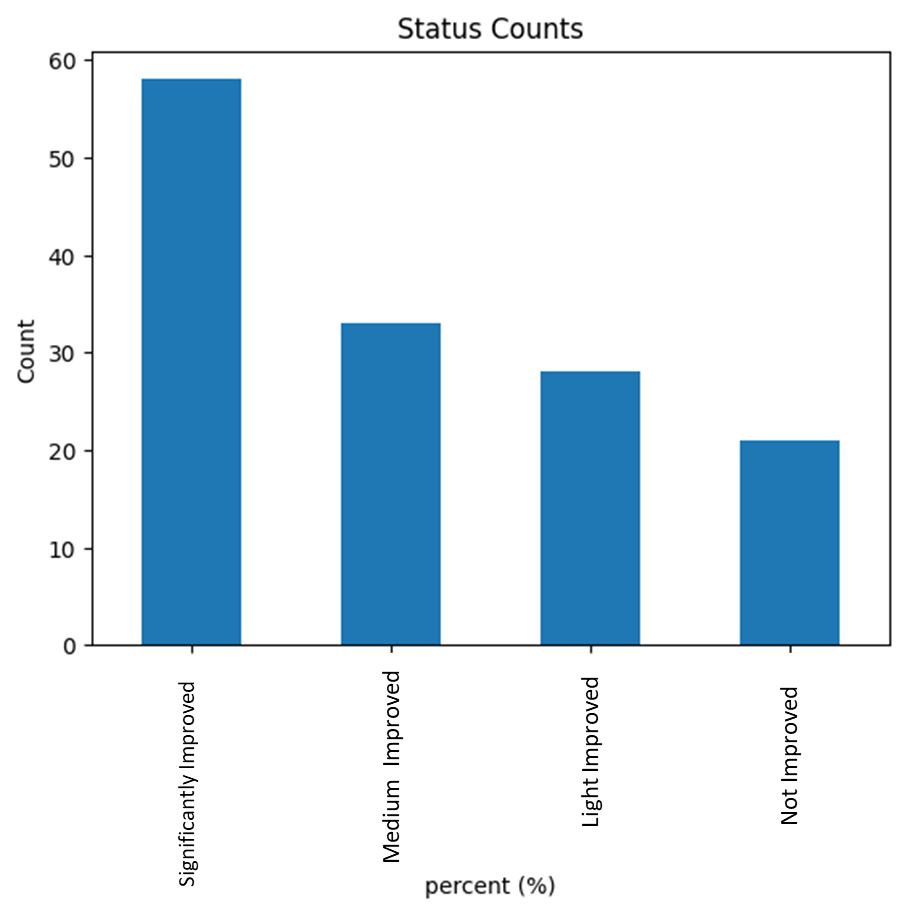


**Figure S6.** HE staining of liver sections from control, MASH, and various treatment groups. (a) Control: Normal liver structure with central veins (CV), portal tracts (PT), and hepatocytes with acidophilic cytoplasm and central vesicular nuclei. (b) MASH: Severe steatosis, hepatocyte ballooning, Mallory-Denk bodies (▲), and significant fibrosis and inflammation. (c) Febuxostat-1.5: Improved liver architecture with some hepatocyte vacuolation. (d) Febuxostat-3: Better preservation of liver structure with reduced steatosis and hepatocyte ballooning. (e) Febuxostat-6: Near-normal liver architecture with minimal steatosis and hepatocyte vacuolation. (f) Amlodipine: Moderate improvement with reduced inflammation and Mallory-Denk bodies. (g) Perindopril: Similar improvements to Amlodipine. (h) Amlodipine & Perindopril combination: Significant reduction in inflammation, ballooning, Mallory-Denk bodies, and fibrosis. (i) Atorvastatin-20: Moderate effects with reduced severe steatosis and mild improvement in other parameters. Scale bar: 20 μm; magnification: ×400. Key: CV (central vein); PT (portal tracts); S (hepatic sinusoids); ↑ (acidophilic hepatocytes); ∆ (hepatocyte ballooning); Elbow arrow (macro-vesicular steatosis); Curved arrow (micro-vesicular vacuolation); Thick arrow (large vacuoles with eccentric nuclei); ▲ (Mallory-Denk bodies); * (cellular infiltration); ♦ (dilated sinusoids); Dashed arrow (lipofuscin).


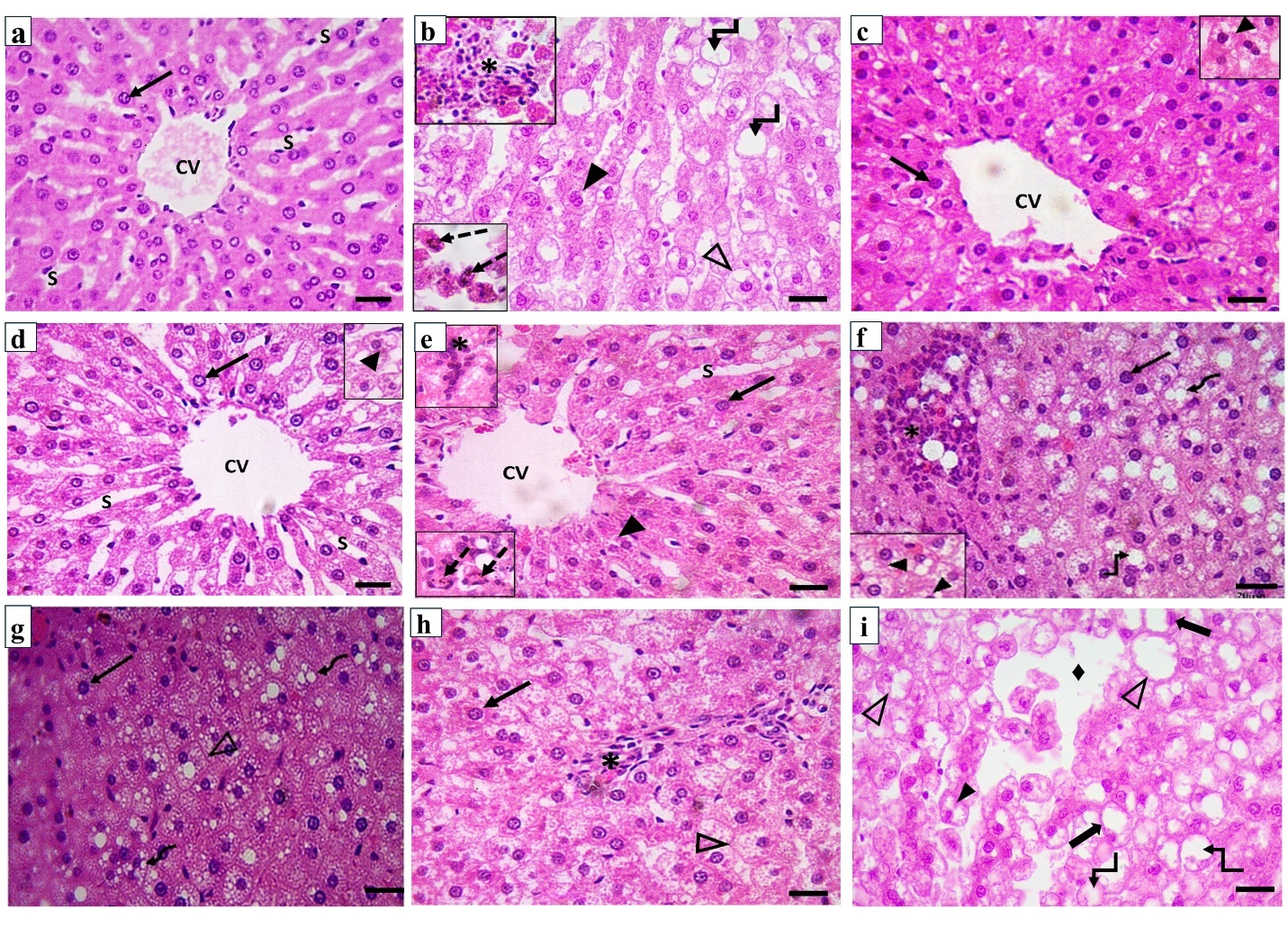


**Figure S7.** photomicrographs of Sirius red-stained liver sections from from control, MASH, and various treatment groups illustrating collagen fiber distribution: around the central vein (CV) (↑), around portal tracts (PT) (▲), pericellular collagen fibers (thick arrow), and bridging fibrosis (elbow arrow). in (a) control, (b) MASH-induced, (c) Febuxostat-1.5 treated, (d) Febuxostat-3 treated, (e) Febuxostat-6 treated, (f) Amlodipine treated, (g) Perindopril treated, (h) Amlodipine & Perindopril combined treatment, and (i) Atorvastatin-20 treated groups. Scale bar = 100 μm; magnification: x100.


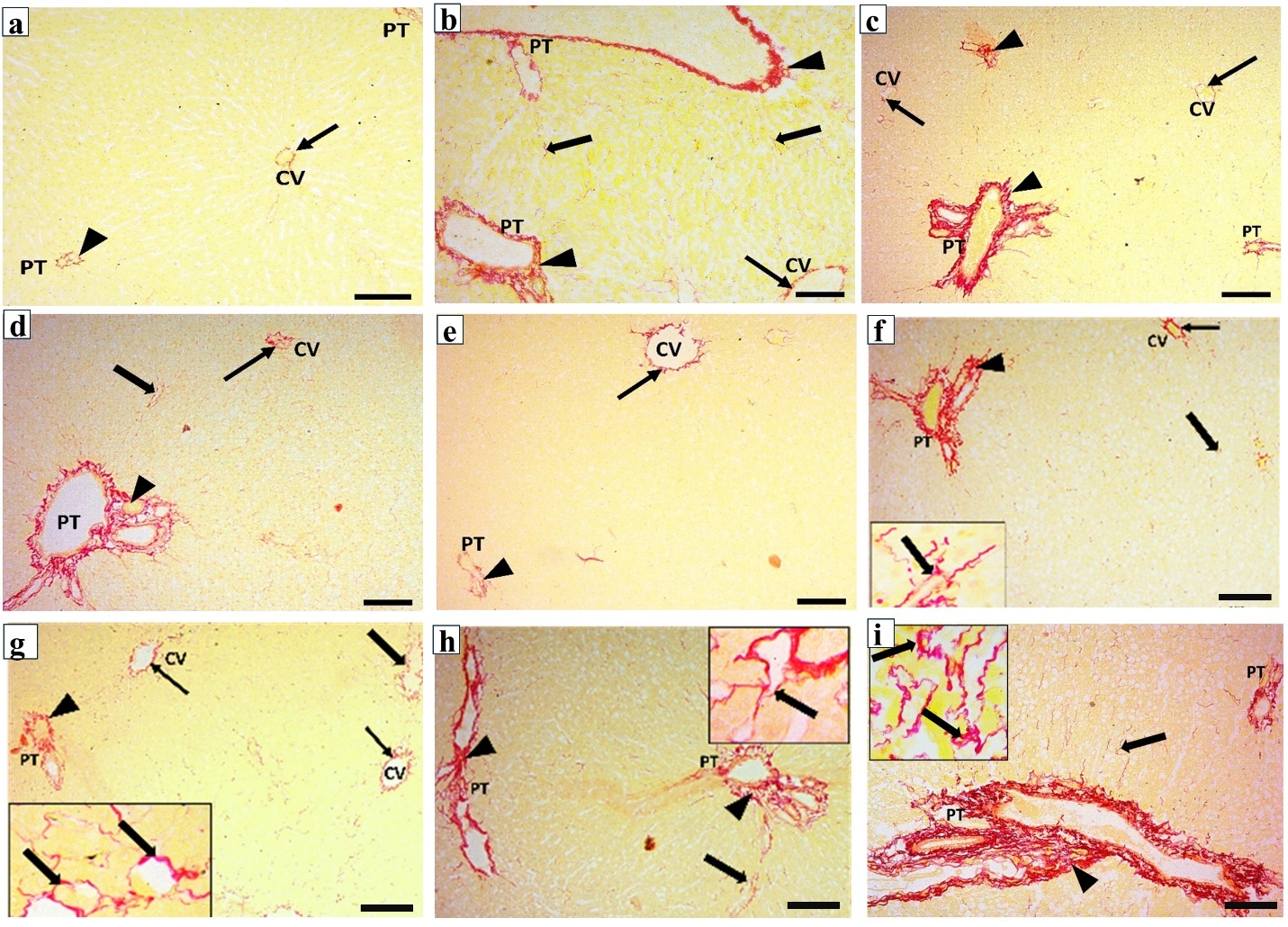

Supplement: Supplementary file 2 — Supplementary Material 2 (DOCX 12.8 MB) [file 13105_2026_1181_MOESM2_ESM.docx]
